# The auxiliary ESCRT complexes provide robustness to cold in poikilothermic organisms

**DOI:** 10.1242/bio.043422

**Published:** 2019-08-14

**Authors:** Miriam Bäumers, Sven Klose, Christian Brüser, Carl Haag, Sebastian Hänsch, Hendrik Pannen, Stefanie Weidtkamp-Peters, Michael Feldbrügge, Thomas Klein

**Affiliations:** 1 Institute of Genetics, Heinrich-Heine-University Düsseldorf, Universitätsstr. 1, 40225 Düsseldorf, Germany; 2 Institute of Microbiology, Cluster of Excellence on Plant Sciences, Heinrich-Heine-University Düsseldorf, Universitätsstr. 1, 40225 Düsseldorf, Germany; 3 Center of Advanced Imaging (CAi), Heinrich-Heine-University Düsseldorf, Universitätsstr. 1, 40225 Düsseldorf, Germany

**Keywords:** ESCRT complexes, ESCRT-III, Cold-sensitivity, Notch signalling, Chmp5, Vps60, Ist1, Auxiliary ESCRTs, *Drosophila*, *Ustilago maydis*

## Abstract

The ESCRT pathway, comprising the in sequence acting ESCRT-0, -I, -II, -III and Vps4 complexes, conducts the abscission of membranes away from the cytosol. Whereas the components of the central ESCRT-III core complex have been thoroughly investigated, the function of the components of the associated two auxiliary ESCRT sub-complexes are not well-understood in metazoans, especially at the organismal level. We here present the developmental analysis of the *Drosophila* orthologs of the auxiliary ESCRTs Chmp5 and Ist1, DChmp5 and DIst1, which belong to the two auxiliary sub-complexes. While each single null mutant displayed mild defects in development, the *Dist1 Dchmp5* double mutant displayed a severe defect, indicating that the two genes act synergistically, but in separate pathways. Moreover, the presented results indicate that the auxiliary ESCRTs provide robustness against cold during development of diverse poikilothermic organisms, probably by preventing the accumulation of the ESCRT-III core component Shrub on the endosomal membrane.

## Abstract (precis)

**Summary:** The analysis of Chmp5 and Ist1, which belong to the two ESCRT auxiliary sub-complexes in *Drosophila*, suggests that these ESCRT proteins provide robustness against cold in diverse poikilothermic organisms.

## INTRODUCTION

Degradation of transmembrane proteins (TMPs) is initiated by their endocytosis from the plasma membrane into early endosomes (EEs, reviewed in Huotari and Helenius, 2011). These EEs mature and eventually fuse with lysosomes where their luminal cargo is degraded by acidic hydrolases. On the way to degradation, TMPs are internalised from the limiting membrane into the lumen of maturing endosomes (MEs) through the generation of intraluminal vesicles (ILVs). ILV formation is necessary to degrade the intracellular domain (ICD) of TMPs, which are in contact with the cytosol in EEs. It is also necessary to terminate signalling by activated signalling receptors, as it physically separates their ICDs from the cytosol. ILVs are constantly formed throughout the life of the ME. Hence, endosomes progressively accumulate more ILVs during maturation and are recognised as multi-vesicular bodies (MVBs) in the transmission electron microscope (TEM).

ILVs are generated by the activity of the evolutionarily conserved endosomal sorting complex required for transport (ESCRT) machinery. Five ESCRT complexes [ESCRT-0 to ESCRT-III and the vacuolar protein sorting 4 (Vps4) complex] act one after the other to first concentrate ubiquitylated cargo in budding zones (ESCRT-0 till -II) and then invaginate the membrane spot as an ILV (ESCRT-III and Vps4) (Henne et al., 2011; Huotari and Helenius, 2011). CHMP4/Vps32/Snf7, termed Shrub in *Drosophila*, is a key player during ILV formation and the core component of the ESCRT-III complex. It belongs to the charged multivesicular body protein (CHMP) family that includes all ESCRT-III core components (Howard et al., 2001). Shrub is recruited to the limiting membrane by the first acting ESCRT-III component Vps20/CHMP6 and forms filamentous homo-polymers on the membrane that, in concert with the AAA ATPase Vps4, perform the abscission reaction (Hurley, 2015). Core ESCRT-III contains three additional CHMP family members. Vps20 initiates the polymerisation of Shrub at the membrane. Vps24/CHMP3 and Vps2/CHMP2 terminate Shrub polymerisation and recruit the Vps4 complex, respectively. Vps4 disassembles the ESCRT-III polymer into monomers, which are re-used in subsequent rounds of ILV formation. Thus, Shrub cycles between a monomeric cytosolic and a polymeric membrane bound form. In *Drosophila*, the loss of ESCRT function causes the ectopic ligand-independent activation of the Notch pathway, as well as prolonged signalling by other signalling pathways, such as the BMP pathway (Thompson et al., 2005; Vaccari et al., 2008).

For the full function of Vps4, auxiliary factors are required. Several of these also belong to the CHMP family and are best characterised in yeast. The yeast data are summarised in the following. The auxiliary factors form two sub-complexes: increased sodium tolerance 1 (Ist1) binds to Doa4-independent degradation 2 (Did2) to facilitate the recruitment of Vps4 to the assembled ESCRT-III complex (Agromayor et al., 2009; Dimaano et al., 2008; Rue et al., 2008), while Vps60 and Vps twenty associated 1 (Vta1) form the second sub-complex that enhances Vps4 activity (Azmi et al., 2006, 2008; Shiflett et al., 2004; Xiao et al., 2008). The auxiliary ESCRTs display a much weaker loss of function phenotype, ranging from no to weak trafficking defects (Agromayor et al., 2009; Dimaano et al., 2008; Rue et al., 2008). In addition to its role in one of the sub-complexes, it has been recently shown that Did2 is involved in long-distance mRNA transport in the fungus *Ustilago maydis* (Haag et al., 2017).

Less information is available about the function of the auxiliary ESCRTs in metazoans. In mice, loss of the Vps60 ortholog CHMP5 causes early embryonic lethality and weak activation of the TGFß-signalling pathway (Shim et al., 2006). Moreover, conditional knockout in osteoclasts revealed that it regulates the activity of NF-κB signalling in these cells (Greenblatt et al., 2015). Human IST1 is also known as overexpressed in lung cancer 1 (OLC1), since it is overexpressed in many tumours, such as lung and colorectal cancer (LIU et al., 2014; Yuan et al., 2008). Furthermore, it has been shown that human IST1 is involved in cytokinesis, where it recruits the AAA ATPase Spastin to sever microtubules (Agromayor et al., 2009; Vietri et al., 2015). In *Drosophila*, loss of *Dchmp5* function in the follicle epithelium results in elongated stalks caused by a degradation defect of Notch and an over-activation of JAK/STAT signalling (Berns et al., 2014). In most other investigations of the auxiliary ESCRTs, Gal4/RNAi mediated depletion was analysed. It is therefore not known whether null mutant situations were analysed. Ubiquitous depletion of CHMP1/Did2 resulted in lethality; depletion during wing development in weak wing margin phenotypes that might be caused by interference with the activity of the Notch pathway (Valentine et al., 2014). A recent publication shows an involvement in long-range signalling of the Hedgehog (Hh) pathway (Coulter et al., 2018; Matusek et al., 2014).

Here, we report the analysis of the function of the two auxiliary ESCRT sub-complexes in *Drosophila* by analysing null mutants of the orthologs of Chmp5 and Ist1 (*Dchmp5* and *Dist1*). We found that mutants of both genes show a cold-sensitive phenotype. Our findings reveal an unexpected role for the auxiliary ESCRTs during development of *Drosophila*, which is to neutralise the deleterious accumulation of Shrub at the endosomal membrane at cold temperatures. The cold sensitivity was also observed for CHMP5/Vps60 mutants of the phytopathogenic fungus *U.* *maydis*. Thus, it appears that the auxiliary ESCRT complexes confer robustness to the ESCRT mediated process against cold in several poikilothermic eukaryotic organisms.

## RESULTS

### Loss of *Dchmp5* function causes a temperature dependent arrest in development

**Fig. 1.**
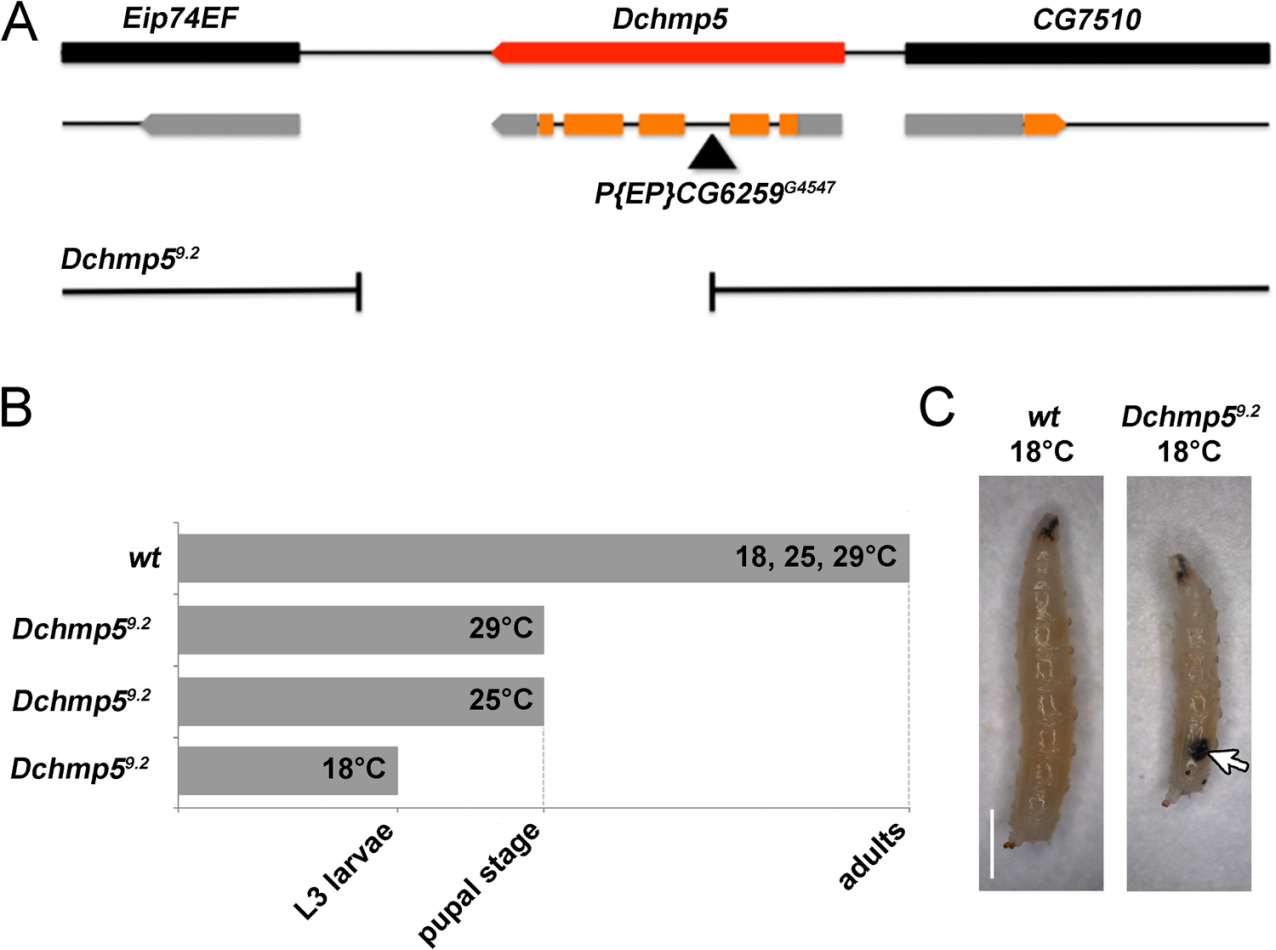
**Loss of *Dchmp5* function causes a temperature-dependent arrest in development.** (A) The genomic region of *Dchmp5* (adapted from FlyBase). For simplification, only the predicted isoform A is shown, exons in orange and UTRs in grey. The black triangle indicates the insertion site of *P(EP)CG6259^G4547^*. In *Dchmp5^9.2^* the coding sequence downstream of exon 2 is deleted. (B) Summary of the time of death of *Dchmp5^9.2^* mutants raised at different temperatures. (C) Wild-type and *Dchmp5^9.2^* mutant larvae of the third instar stage constantly raised at 18°C. The rare *Dchmp5^9.2^* mutant larvae develop melanotic tumours (arrow, 100% penetrance, *n*=6). Scale bar: 1 mm.

Since no mutant alleles of *Dchmp5* were available in *Drosophila melanogaster* at the start of our analysis, we generated alleles by imprecise P-element excision of *P(EP)CG6259^G4547^*, which is inserted in the second intron of *Dchmp5* (*CG6259*) (Fig. 1A). Subsequent molecular characterisation indicated that the isolated *Dchmp5^9.2^* is probably a null allele, since the coding sequence downstream of exon 2 is deleted (Fig. 1A). The predicted truncated protein lacks 154 of 226 amino acids of DChmp5. The missing part includes most of the Snf7 domain, which is an essential hallmark of all CHMP family members, as well as the MIT-interacting motif 1 (MIM1) and MIM2 domains that mediate the interaction with Vta1/LIP5 and Vps4 (Azmi et al., 2008). Complementation tests ruled out that the adjacent downstream-located gene *Eip74EF* is affected by the deletion.

*Dchmp5^9.2^* homozygosity caused pupal lethality at 25°C and 29°C (Fig. 1B). By keeping the stocks in our stock collection at 18°C, we noticed an enhancement of the mutant phenotype (Fig. 1B). At this low temperature, only a few larvae developed to the third instar stage and did not pupariate. These rare escapers contained melanotic tumours (100% penetrance, *n*=6; Fig. 1B,C, arrow). To confirm that cold sensitivity is a general consequence of the loss of function of *Dchmp5* and not a specific characteristic of *Dchmp5^9.2^*, we analysed an independently induced null allele termed *Df(3L)d08528-f02548* (Berns et al., 2014) and observed a similar cold sensitivity.

### Regulation of wing disc growth and signalling by Dchmp5

**Fig. 2.**
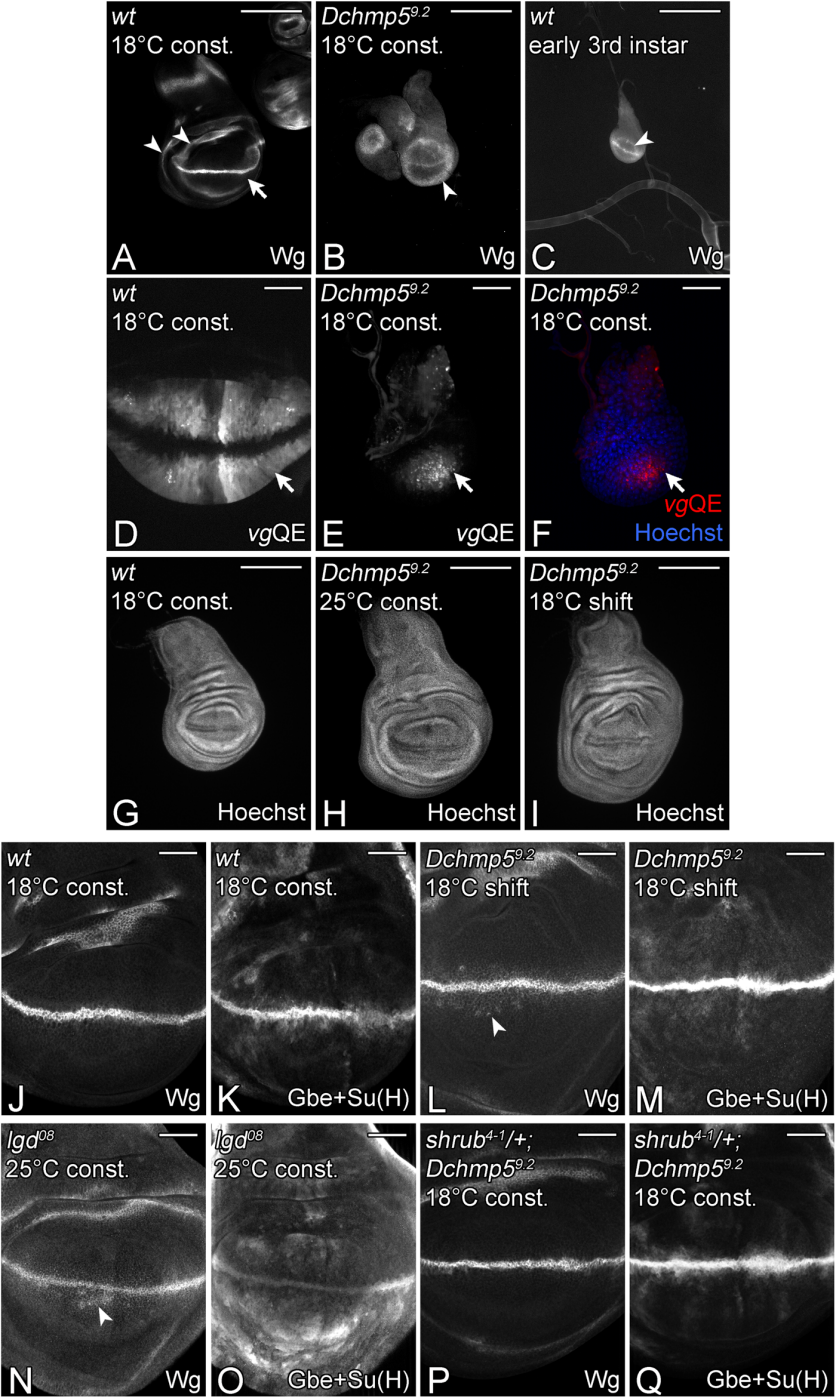
**Regulation of cell proliferation and Notch signalling by DChmp5.** (A) Wild-type late third instar wing imaginal discs raised constantly at 18°C. The phenotype is the same at 25 and 29°C. The expression pattern of Wg is shown. The arrowheads point to the inner and outer ring-like expression domains of Wg that are independent of Notch signalling. The stripe-like, Notch-dependent domain is straddling the D/V-boundary (arrow). (B) *Dchmp5^9.2^* mutant third instar wing imaginal discs constantly kept at 18°C. The phenotype of the wing disc resembles that of a wild-type disc at the early third larval instar stage depicted in C. Only one ring-like expression domain of Wg is present and the disc is small (arrowhead in B,C). (D) Expression pattern of the *vgQE* enhancer in the wing pouch of a wild-type third instar larval stage constantly raised at 18°C. The expression of the *vgQE* marks the developing wing pouch with exception of the D/V boundary. The arrow highlights the expression domain of the *vgQE*. (E,F) In contrast, in a disc homozygous mutant for *Dchmp5^9.2^* constantly kept at 18°C, the *vgQE* is expressed only in a region around the intersection of the anteroposterior (A/P) and D/V boundaries (arrow). (G–I) Hoechst staining of wild-type (G) and *Dchmp5^9.2^* mutant wing discs (H,I), either constantly kept at 25°C (H), or shifted to 18°C throughout the third instar stage (I). The comparison with G reveals the increased size of the shifted mutant disc. This phenotype is fully penetrant. (J,K) Expression of Wg and Gbe+Su(H)-lacZ in a wild-type late third instar disc. (L,M) Expression of Wg and Gbe+Su(H)-lacZ in a *Dchmp5^9.2^* mutant disc shifted to 18°C throughout the third instar stage. The arrowhead in L highlights the weak ectopic expression of Wg in single cells near the D/V boundary. (M) This weak ectopic Notch activation is also confirmed by the more sensitive Notch activity reporter Gbe+Su(H). (N,O) A disc homozygous for the hypomorphic *lgd^08^* allele. It shows a similar weak ectopic expression of Wg (N, arrowhead) and Gbe+Su(H) (O). (P,Q) Late third instar wing disc of a *Dchmp5^9.2^* mutant fly constantly raised at 18°C, which is also heterozygous for the null allele *shrub^4-1^*. The ectopic expression of Wg (P) and Gbe+Su(H) (Q) is lost (compare with L,M). Scale bars: (A–C,G–I) 200 µm; (D–F,J–Q) 50 µm. At least ten wing imaginal discs were analysed for each genotype.

The wing imaginal discs of *Dchmp5*^9.2^ mutant animals were very small and the expression pattern of the Notch target Wingless (Wg) resembled that of early third instar discs of wild-type flies, since only one instead of two ring-like domains in the proximal wing anlage were present (Fig. 2A–C; arrowheads). Moreover, the expression domain of the vestigial Quadrant (*vg*QE) enhancer, was also similar to that of early third instar wing imaginal discs of the wild type (Fig. 2D–F). These findings indicate that *Dchmp5* mutant flies raised in cold conditions arrest their development during the early third instar larval stage.

We could avoid the developmental arrest of the mutant flies by conducting temperature shifts (ts), in which the *Dchmp5^9.2^* mutant flies were kept at 25°C until the end of the second instar larval stage and then shifted to 18°C until the end of the third instar larval stage. The shifted wing discs were larger than those kept constantly at 25°C and also larger than discs of the wild type (Fig. 2G–I). In addition, the Notch target Wg was ectopically expressed in single cells close to the dorso-ventral compartment boundary (D/V-boundary), suggesting a slight ectopic activation of the Notch pathway (Fig. 2J,L). The ectopic Notch activation was confirmed by the more sensitive Notch activity reporter Gbe+Su(H) (Furriols and Bray, 2001; Housden et al., 2012; Fig. 2K,M). The extent of ectopic Notch activation was comparable to that observed in the hypomorphic *lgd^08^* mutant where the Notch pathway is also ectopically activated (Gallagher and Knoblich, 2006; Fig. 2N,O). Ectopic activation of Notch in the wing disc, e.g. as observed in *lgd* mutant discs, induces over-proliferation of disc cells (Doherty et al., 1997; Jaekel and Klein, 2006; Speicher et al., 1994). The similarity of the *Dchmp5^9.2^* mutant phenotype with that of the hypomorphic *lgd^08^* allele suggests that the increase in size of the shifted *Dchmp5^9.2^* mutant disc is probably the result of the observed ectopic activation of the Notch pathway.

### *Dchmp5* cells have enlarged endosomes that accumulate transmembrane proteins, such as Notch and Delta

**Fig. 3.**
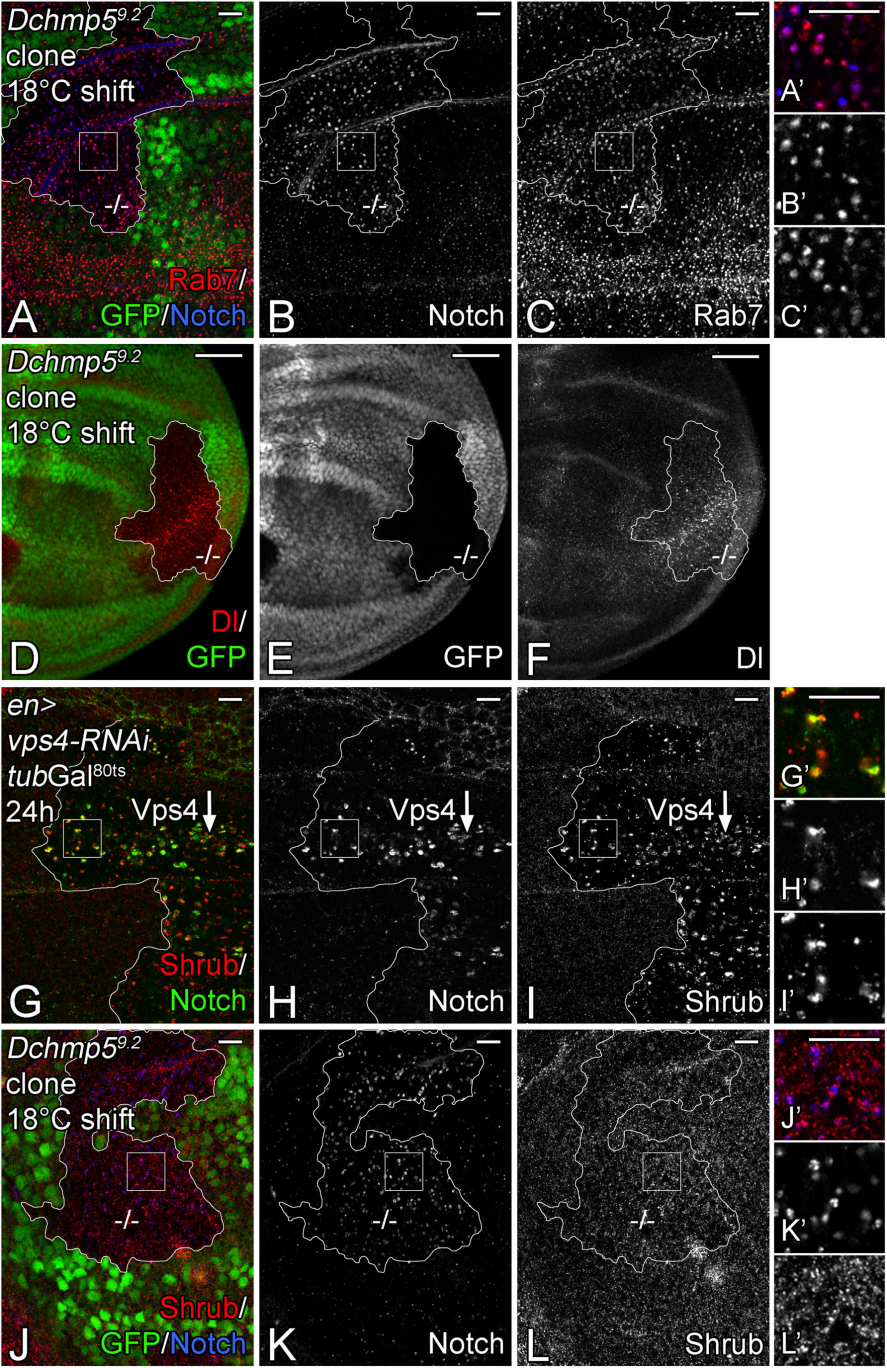
**Loss of *Dchmp5*** **results in an accumulation of** **Notch and Delta in vesicles, but does not affect the distribution of Shrub.** (A–F) Clonal analysis of *Dchmp5^9.2^* in third instar larval wing imaginal discs shifted to 18°C during the third instar larval stage. Clones are labelled by the absence of GFP and outlined in white. *Dchmp5^9.2^* mutant cells contain enlarged Notch and Dl-positive vesicles. The presence of Rab7 identifies them as MEs. Magnification of the square regions in A–C are depicted in A′–C′. (G–I) Expression of *vps4*-RNAi in the posterior compartment for 24 h using a combination of *en*Gal4 and *tubulin*Gal80^ts^ (*tub*Gal80^ts^) results in accumulation of Shrub on Notch-positive MEs. The enGal4 expression boundary is outlined in white. Magnification view of the square regions in G–I are shown in G′–I′. (J–L) Loss of *Dchmp5* function affects only the distribution of Notch, but not of Shrub. In contrast to the depletion of Vps4, no clear accumulation of Shrub on Notch-positive MEs can be observed (compare with G–I). Clonal area is labelled through the absence of GFP and outlined in white. Magnification image of the square areas in J–L are shown in J′–L′. Scale bars: (A–C,G–L) 10 µm; (D–F) 50 µm. At least ten wing imaginal discs were analysed for each genotype.

We used discs bearing *Dchmp5^9.2^* mutant clones, shifted to 18°C throughout the third instar larval stage, for further analysis of the *Dchmp5* mutant endosomal phenotype. We found that TMPs, such as Notch and Delta accumulated in large vesicles in cells of *Dchmp5^9.2^* mutant clones (Fig. 3A–F). These enlarged vesicles were positive for the endosomal organiser Rab7 and therefore are MEs (Fig. 3A–C).

In yeast, Vps4 is required for the disassembly of the homo-polymer of ortholog Snf7 at the endosomal membrane (Babst et al., 1998). Consequently, reduction or loss of *vps4* function causes accumulation of Snf7 at the endosomal membrane (Babst et al., 1998). Since Chmp5 is required for the full activity of Vps4, its loss of function might cause a similar accumulation of Shrub in *Drosophila* (Azmi et al., 2008; Rue et al., 2008). We raised an antibody against Shrub to test this prediction. The specificity of the antibody was confirmed in a western blot and RNAi depletion and overexpression experiments (see the Materials and Methods and Fig. S1). In accordance with a previous report, Shrub was detectable throughout the cytosol in a punctate distribution without any obvious association with Notch-positive endosomes in wild-type disc cells (Sweeney et al., 2006; Fig. 3I,L; Fig. S1B–F). As observed for Snf7 in yeast, Shrub accumulated on endosomes in Vps4 depleted disc cells (Fig. 3G–I). In contrast, the distribution of Shrub was unaffected in *Dchmp5^9^*^.2^ mutant imaginal disc cells induced by clonal analysis. No specific association with Notch-positive endosomes was observed (Fig. 3J–L). Thus, the loss of *Dchmp5* function has little effect on the subcellular distribution of Shrub and the activity of Vps4.

### MVB size is regulated by DChmp5 in a temperature dependent manner

**Fig. 4.**
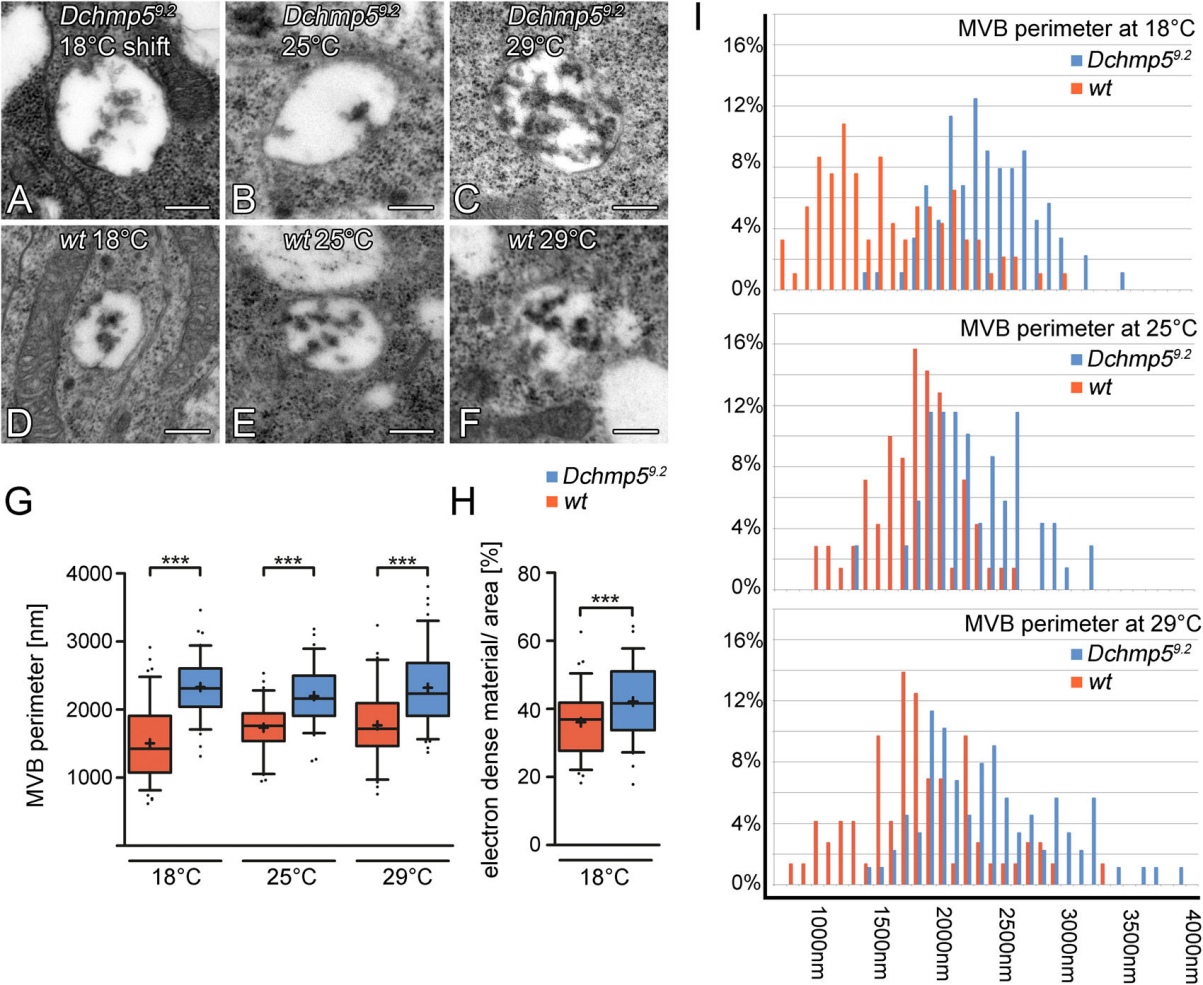
**The MVB size is affected by loss of *Dchmp5* function in a temperature dependent manner.** Representative TEM pictures of MVBs of *Dchmp5^9.2^* mutant (A–C) and wild-type wing disc cells (D–F) at 18°C, 25°C and 29°C. In comparison with the wild type the overall morphology of the *Dchmp5^9.2^* mutant MVBs seems to be unchanged. (G–I) Statistical analysis of MVBs of wild-type (red) and *Dchmp5^9.2^* mutant (blue) wing disc cells at different temperatures. (G) The average perimeter of *Dchmp5^9.2^* mutant MVBs is significantly increased in comparison to the wild type at all temperatures (18°C: wt *n*=92; *Dchmp5^9.2^ n*=88; *t*-test: *P*<0.001) (25°C: wt *n*=70; *Dchmp5^9.2^ n*=69; *t*-test: *P*<0.001) (29°C: wt *n*=72; *Dchmp5^9.2^ n*=88; *t*-test: *P*<0.001) (box plots: whiskers: 5–95 percentile; mean shown as ‘+’). (H) Pixel wise quantification of electron dense material per area within the lumen of wt and *Dchmp5^9.2^* mutant MVBs to measure ILV formation. For details of measurement, see the Materials and Methods. *Dchmp5* mutant MVBs contain significantly more ILVs (18°C: wt *n*=78; *Dchmp5^9.2^ n*=66; *t*-test: *P*<0.001) (box plots: whiskers: 5–95 percentile; mean shown as ‘+’). (I) Abundance of ME/MVBs in disc cells grouped in different size classes (100 nm steps). The loss of *Dchmp5* causes an overall shift in the distribution to larger size classes. Moreover, *Dchmp5^9.2^* mutant cells have MVBs of larger size that do not exist in wild-type cells at temperatures analysed. Scale bars in TEM images: 250 nm.

In the transmission electron microscope (TEM), the overall appearance of *Dchmp5^9.2^* mutant MEs/MVBs was normal irrespective of the temperature (Fig. 4A–F). However, size measurements revealed that they were larger than their wild-type counterparts at all temperatures (Fig. 4G). The difference in size between wild-type and mutant MEs was most severe when the disc cells were shifted to 18°C. Note, that the wild-type MVBs are on average smaller at 18°C than at higher temperatures, while the size of the mutant MVBs is on average similar at all temperatures. This suggests that DChmp5 is required to exert a morphological change to adapt to cold. Classification of MVBs according to their size, revealed the presence of larger size classes in *Dchmp5^9.2^* mutant cells that did not exist in wild-type cells (Fig. 4I).

Although the mutant MEs/MVBs contained ILVs, it is possible that their number is reduced compared to wild-type MEs, as the activity of the ESCRT machinery is affected. To quantify ILV formation, we measured the pixel content of the electron dense material within wild-type and *Dchmp5^9.2^* mutant MVBs (see the Materials and Methods). Surprisingly, the MVBs of mutant cells contained significantly more dense material in their lumen, suggesting that ILV formation is slightly enhanced rather than reduced (Fig. 4H). Altogether these results indicate that, in contrast to yeast, the loss of *Dchmp5* function causes a morphological detectable endosomal phenotype in *Drosophila*.

The loss of function of ESCRT-I, -II and -III core components results in the loss of epithelial polarity of the imaginal disc cells. We therefore monitored the polarity of shifted *Dchmp5^9.^*^2^ mutant cells (Fig. S2). Judged by the normal localisation of the zonula adherens (ZA) core component DE-Cadherin (DE-Cad), as well as Discs large (Dlg), which localises to the basolateral membrane domain, no polarity defect was detected (Fig. S2A–C). In addition, the appearance of the ZA and septate junctions (SJs) looked normal in the TEM, even if the flies were constantly raised at 18°C (Fig. S2D).

### Overexpression of DChmp5 has no obvious impact on endosome maturation

We generated and expressed a HA-tagged version of DChmp5 with the Gal4 system to monitor its subcellular localisation and effects of DChmp5 overexpression on endosome maturation (Fig. S3A). HA-DChmp5 was able to suppress the endosomal phenotype observed in *Dchmp5^9.2^* mutant cell clones (Fig. S3B–I), indicating that the construct is functional. Similar to Shrub, HA-DChmp5 was evenly distributed throughout the cytosol with no obvious association with endosomes (Fig. S3D,H,L). Overexpression of HA-DChmp5 in the posterior compartment with *engrailed*GAL4 (*en*Gal4) (Fig. S3B–I), or strong ubiquitous overexpression with *arm*GAL4 (not shown), had no detectable effect on wing imaginal disc morphology, size of the Notch-positive endosomes or the activity of the Notch pathway, measured by expression of Wg (Fig. S3J–M).

### Reduction of *shrub* activity suppresses the phenotype of *Dchmp5* mutants

**Fig. 5.**
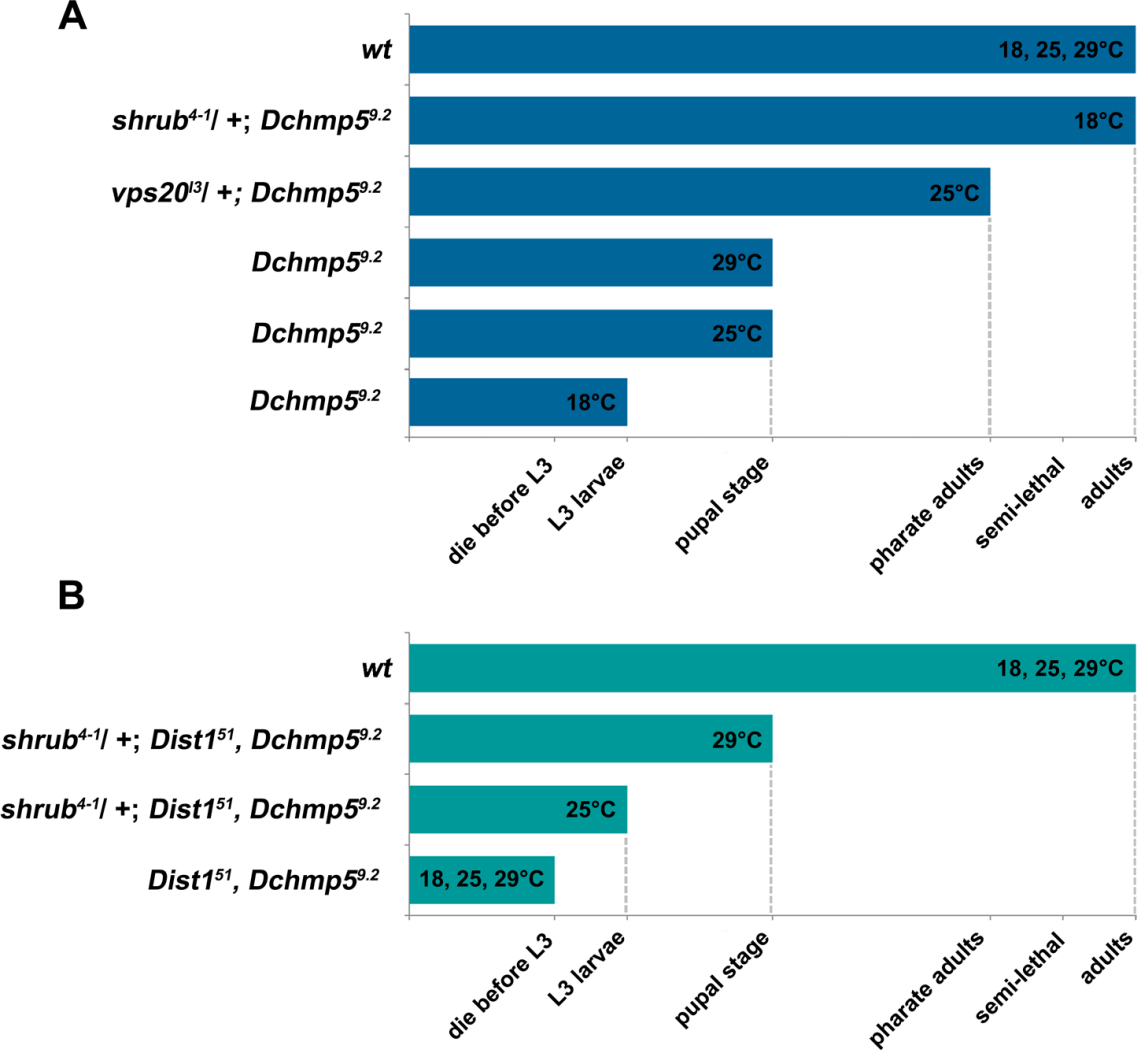
**Genetic interactions of *shrub* with *Dchmp5* single and *Dchmp5 Dist1* double mutants revealed by the time of death during development.**

In yeast Vps60/Chmp5 is required for the activity of Vps4. Since Vps4 is in turn required for the functionality of Shrub, we investigated whether DChmp5 influences the activity of Shrub by genetic interaction experiments. In contrast to what we expected, loss of one functional copy of *shrub* (genotype: *shrub^4-1^*/+; *Dchmp5^9.2^/ Dchmp5^9.2^*) rescued *Dchmp5^9.2^* mutants to the adult stage (Fig. 5A). The adult flies displayed no obvious defects, even if continuously raised at 18°C. Hence, reducing the activity of *shrub* compensates for the loss of *Dchmp5* function. Importantly, the Notch induced ectopic expression of Wg, observed in shifted *Dchmp5*^9.2^ mutant discs, was abolished (Fig. 2P,Q, compare with L,M). This finding indicates that the ectopic activation of the Notch pathway was induced by a defect in the activity of the ESCRT machinery.

We have previously shown that the loss of one functional copy of *shrub* reduces the activity of the ESCRT machinery to a level that causes a weak mutant phenotype during oogenesis in *Drosophila* (Morawa et al., 2015). Therefore, we expected that reducing the activity of *shrub* in addition to loss of function of *Dchmp5* should synergistically reduce the function of ESCRT machinery and, hence, lead to a stronger mutant phenotype, but not to the weaker phenotype observed. The result is compatible with our observation that *Dchmp5* mutant MEs contain more ILVs (Fig. 4H). It appears that loss of *Dchmp5* function results in an over-activity of Shrub.

One possible reason why Shrub is deleterious in *Dchmp5^9.2^* mutant cells is that it accumulates on the endosomal membrane. Vps20 is required for the recruitment of Shrub monomers to the endosomal membrane and the initiation of its polymerisation. We reasoned that the loss of one functional copy of *vps20* should reduce the accumulation of Shrub at the endosomal membrane and therefore lead to a similar rescue of *Dchmp5^9.2^* mutants. Indeed, we found that also removal of one functional copy of *vps20* resulted in a rescue of *Dchmp5^9.2^* mutants at 25°C, but in this case only up to the pharate adult stage (genotype: *vps20^I3^*/+; *Dchmp5^9.2^/Dchmp5^9.2^*; Fig. 5A). This finding supports the notion that the accumulation of Shrub at the endosomal membrane in *Dchmp5* mutants is deleterious for development.

### Loss of *Dist1* function results in lethality at low temperatures

To inactivate the other auxiliary ESCRT sub-complex, consisting of Ist1 and Did2, we generated alleles of *Dist1* (*CG10103*) through imprecise excision of the P-element insertion *P(EP)CG10103^G6550^*, which is inserted in the 5′ UTR of *CG10103* (Fig. S4A). We obtained *Dist1^51^*, an allele where 972 bp of the coding region including the transcriptional start is deleted (Fig. S4A). Therefore, *Dist1^51^* is probably a null allele. Homozygous *Dist1^51^* flies developed into sterile adults at 29°C (both sexes), but died as pharate adults if raised at 18°C (Fig. S4B). At 25°C, loss of function of *Dist1^51^* was semi-lethal. No obvious pattern defects were observed at any temperature. Hence, loss of *Dist1* function also caused a cold-sensitive phenotype.

We did not observe ectopic activation of the Notch pathway (Fig. S4C–F). Clonal analysis revealed that *Dist1^51^* mutant cells had normal Notch- and Rab7-positive endosomes whose size and appearance were comparable to that of their wild-type neighbours (Fig. S4G–I). The distribution of Shrub was normal in cells of mutant wing imaginal discs (Fig. S4J,K). TEM analysis revealed that the size and morphology of *Dist1^51^* mutant MVBs was normal (Fig. S4L,M). Hence, the phenotype of *Dist1^51^* mutants is considerably weaker than that of *Dchmp5^9.2^* mutants.

### *Dist1* and *Dchmp5* act in distinct, but synergistically acting pathways

**Fig. 6.**
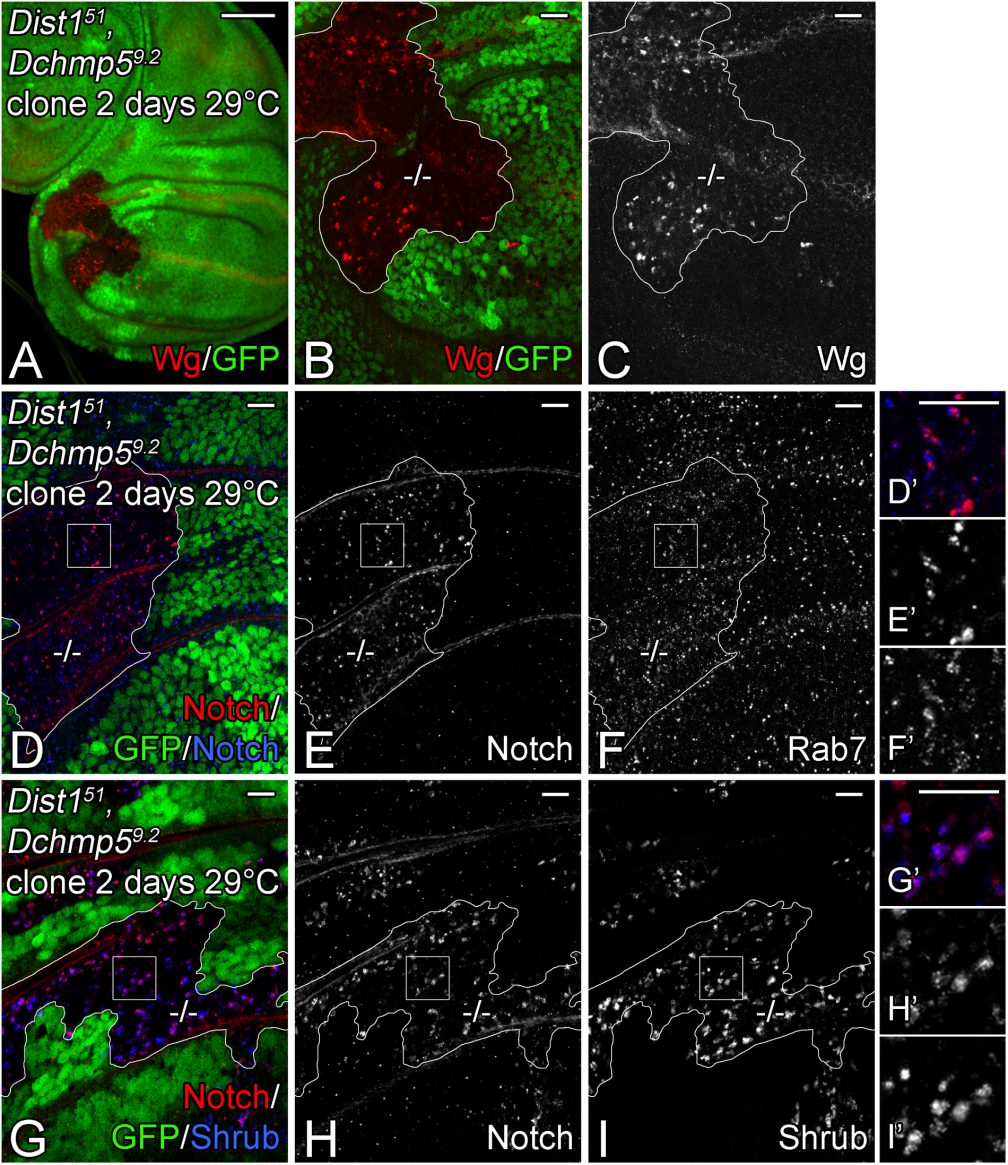
**Concomitant loss of function of *Dchmp5* and *Dist1* results in an accumulation of Shrub at the endosomal membrane.** (A–I) Clonal analysis of *Dist1^51^ Dchmp5^9.2^* double mutant cells in imaginal discs raised at 29°C with the second protocol described in the text. Clones are labelled through absence of GFP and outlined in white. Magnification of the square regions in D–F and G–I are shown in D′–F′ and G′–I′, respectively. (A–C) Wg accumulates in enlarged vesicles in the mutant cells. (D–F) These enlarged vesicles are Rab7-positive MEs that also contain Notch. (G–I) Moreover, Shrub accumulates on the double mutant MEs. Scale bars: (A–C) 50 µm; (D–I) 10 µm. At least ten wing imaginal discs were analysed for each genotype.

**Fig. 7.**
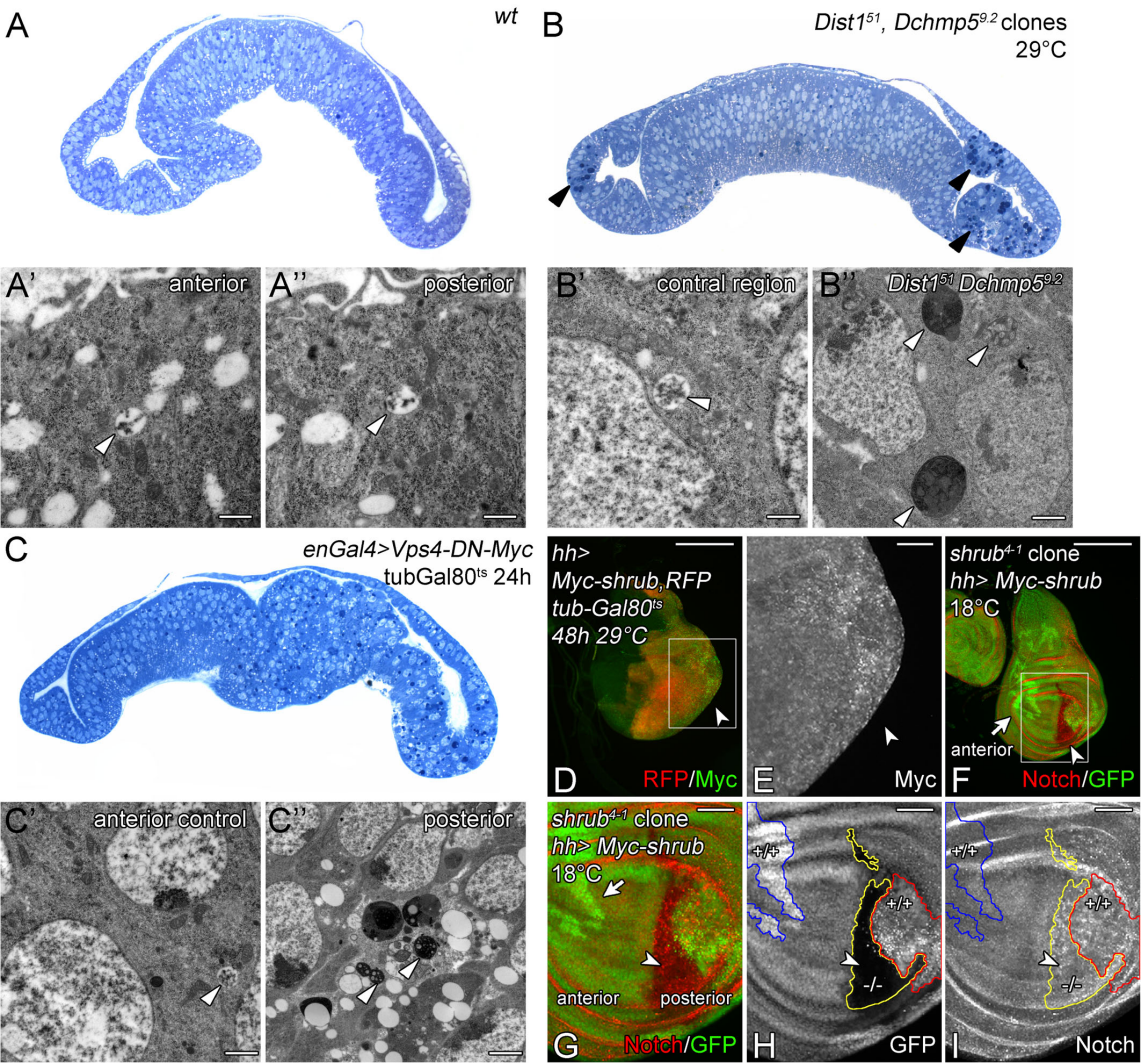
**Ultra-structural analysis of *Dist1^51^ Dchmp5^9.2^* double mutant cells.** (A–C) Transverse semi-sections of analysed wing imaginal discs (anterior to the left). (A–A″) Wild-type wing imaginal disc and the corresponding MEs that are detected in the TEM (A′ and A″, arrowheads). (B–B″) Wing imaginal disc raised at 29°C that bears *Dist1^51^ Dchmp5^9.2^* double mutant clones. The *Dist1^51^ Dchmp5^9.2^* double mutant cells separate from the tissue (B, arrowheads) and contain dark material. In the TEM, MVBs are only observable in cells of the control regions (B′, arrowhead), but not in *Dist1^51^ Dchmp5^9.2^* double mutant cells. The double mutant cells contain only electron-dense enlarged class E-like structures (B″, arrowheads). (C–C″) Wing imaginal disc where *UAS Vps4-DN-Myc* was expressed in the posterior compartment for 24 h using a combination of *en*GAL4 and *tub*GAL80^ts^. In the anterior control region MEs were detected (C′, arrowhead). In posterior cells that over-express Vps4-DN-Myc the MEs were also replaced by large class E-like structures (C″, arrowhead). Scale bars in TEM images: 250 nm. For ultra-structural analysis serval MVBs in at least three wing imaginal discs were analysed (*n*≥3). (D–I) Consequences of Shrub overexpression. (D,E) Expression of Myc-Shrub in the posterior compartment (RFP-positive) for 48 h results in over-proliferation of posterior disc cells (arrowhead). (E) Magnification of the posterior area of the disc shown in (D, white box). Myc-Shrub accumulates on MEs that are seen as Myc-positive punctate structures. (F–I) Disc bearing *shrub* clones, labelled through the absence of GFP. Simultaneously, Myc-Shrub is expressed in the posterior compartment with *hh*Gal4 at 18°C. (G–I) Magnification of the wing area of the disc shown in F (white box). Orphan wild-type clones in the anterior (outlined in blue in H,I and marked by an arrow in F,G) indicate that complete loss of *shrub* function is cell lethal. In contrast, a large *shrub* mutant clone can be observed in the posterior compartment where Myc-Shrub is expressed. Thus, in the posterior compartment where Myc-Shrub is present, the mutant cells survive, indicating that the construct is functional. Note, that enlarged Notch-positive MEs are present in wild-type (red outlined) and *shrub* mutant (yellow outlined) areas, indicating that a strong overexpression of functional Shrub results in a change in ME morphology. Scale bars: (D,F) 200 µm; (E,G–I) 50 µm. At least ten wing imaginal discs were analysed for each genotype.

In yeast, Ist1 and Chmp5 are involved in recruitment of Vps4 to the assembled ESCRT-III complex and also in its subsequent activation (Rodahl et al., 2009; Rusten et al., 2007). Since, the loss of function of *vps4* in *Drosophila* causes a much stronger phenotype than the individual loss of *Dchmp5* and *Dist1*, we analysed the phenotype of their combined loss, which is synonymous with the loss of function of both auxiliary sub-complexes. We found that *Dist1^51^ Dchmp5^9.2^* double mutants died before reaching the third instar larval stage at 18°C, 25°C and 29°C (Fig. 5B). Hence, the phenotype is more severe than that of the single mutants. Clonal analysis revealed that also the phenotype of *Dist1^51^ Dchmp5^9.2^* double mutant cells is cold sensitive: at 18°C, no double mutant clones were obtained, indicating that the concomitant loss of function of both genes causes cell lethality at cold temperature. We obtained clones if the flies where kept at higher temperatures by the following two protocols: (1) induction of clones 3 days after egg laying (ael), followed by a shift from 25°C to 29°C for 2 days (Fig. 6). (2) Induction of clones 2 days ael followed by a shift from 25°C to 29°C for 3 days. In this case the mutant clones bulged out of the rest of the disc epithelium (see transverse semi-section Fig. 7B, arrowheads). In both cases, the *Dist1^51^ Dchmp5^9.2^* double mutant cells possessed dramatically enlarged endosomes that contained Wg, Notch and Rab7 (Fig. 6A–F). Importantly, as in the case of depletion of *vps4* function, Shrub accumulated at the endosomal membrane, which was not observed in each single mutant (compare Fig. 6G–I with Fig. 3J–L and Fig. S4J,K). Hence, the concomitant loss of function of *Dist1* and *Dchmp5* results in a failure to remove Shrub from the membrane, suggesting that the function of Vps4 is impaired. In further support of this notion, it has been reported that clones mutant for *vps4* also bulge out of the disc epithelium as observed here for the double mutant clones (Rodahl et al., 2009). Interestingly, we did not observe ectopic activation of the Notch pathway in the double mutant clones (not shown). An explanation for this, at first glance, surprising finding is provided in the discussion.

The fact that *Dist1^51^ Dchmp5^9.2^* double mutant clones bulged out of the imaginal disc tissue enabled us to unambiguously identify and analyse these clones in the TEM (Fig. 7A,B). In contrast to the surrounding wild-type cells, we failed to detect clear MVBs in the double mutant cells in the TEM (compare Fig. 7A–A″ with B–B″). Instead, large vesicles containing membranous structures and electron dense material within their lumen were present (Fig. 7B″, arrowheads). These vesicles resembled the classE compartment described for loss of ESCRT function in yeast, *Drosophila* and mammalian cells (Doyotte et al., 2005; Klämbt, 1993; Maruzs et al., 2015; Raymond et al., 1992). We observed a similar phenotype in cells that expressed a dominant negative variant of Vps4 (Vps4-DN-Myc) in the posterior compartment for 24 h using *hh*GAL4 (Fig. 7C–C″). The similarity of the phenotype of *Dchmp5 Dist1* double and *vps4* single mutants supports the notion that the concomitant loss of function of the two auxiliary ESCRT complexes results in a strong inactivation of Vps4. Since *Dchmp5^9.2^* and *Dist1^51^* are null alleles, the produced synergistic phenotype indicates that the two genes and the corresponding sub-complexes mediate independent functions/processes that together regulate the activity of Vps4. Otherwise, the double mutant phenotype should resemble that of the single mutants.

In contrast to loss of ESCRT-III function, we found that the apico-basal polarity in the obtained double mutant cells was not affected. The ZA component DE-Cad, as well as the baso-lateral membrane component Dlg were localised normally and the ZA had a normal appearance in the TEM (Fig. S2I–L). It is possible that polarity defects would be present at lower temperatures, if the double mutant cells would survive.

### Accumulation of Shrub on the endosomal membrane is deleterious for cells

Next, we asked whether the reduction of *shrub* activity results in a suppression of also the *Dist1^51^ Dchmp5^9.2^* double mutant phenotype, as we have observed for *Dchmp5^9.2^* single mutants. Indeed, we found that the loss of one functional copy of *shrub* allowed the development of the *Dist1^51^ Dchmp5^9.2^* double mutants until the pupal stage at 29°C and to the third instar larval stage at 25°C, while the double mutants never reach the third instar larval stage in presence of two functional copies of *shrub* (Fig. 5B). Hence, the results confirm the conclusion that accumulation of Shrub at the ME is deleterious in the cold in the absence of the activity of the auxiliary ESCRT complexes.

To gain further support for this notion, we analysed the consequences of overexpression of an N-terminal Myc tagged Shrub variant (Myc-Shrub) in the wing imaginal disc. To test whether Myc-Shrub is functional, we induced *shrub* null mutant clones in wing discs where Myc-Shrub is expressed only in the posterior compartment with *hh*Gal4. The construct was expressed at 18°C, where Gal4 is less active, to allow development of the animals to the pupal stage. *shrub* mutant clones have been shown to survive poorly in the wing (Schneider et al., 2013). In agreement with this, we found only orphan wild-type twin clones in the anterior control compartment where Myc-Shrub is not expressed (Fig. 7F,G, arrow and H,I outlined in blue). In contrast, large *shrub* mutant clones were observed in the posterior compartment where Myc-Shrub was expressed, showing that Myc-Shrub can replace the function of endogenous Shrub (Fig. 7F,G, arrowhead and H, I, outlined in yellow). This is the first report of a tagged Shrub/Chmp4 protein that is functional *in vivo*. Importantly, the overexpression of Myc-Shrub with *hh*Gal4 caused the formation of enlarged Notch-positive endosomes in wild-type as well as the rescued *shrub* mutant cells (Fig. 7H–I, outlined in red and yellow, respectively). Moreover, expression of the construct for 48 h induces changes in disc morphology because of over-proliferation of the disc cells and also increased cell death. In addition, Myc-Shrub accumulated at enlarged endosomes (Fig. 7E,I). The phenotype of Shrub overexpression resembles that of *shrub* loss of function (Vaccari et al., 2009; Fig. 7D; arrowhead, not shown). Thus, the overexpression of a functional Shrub protein accumulates at the endosomal membrane and is deleterious for the organism.

### Loss of *vps60*/*chmp5* function causes a cold-sensitivity phenotype in *U. maydis*

**Fig. 8.**
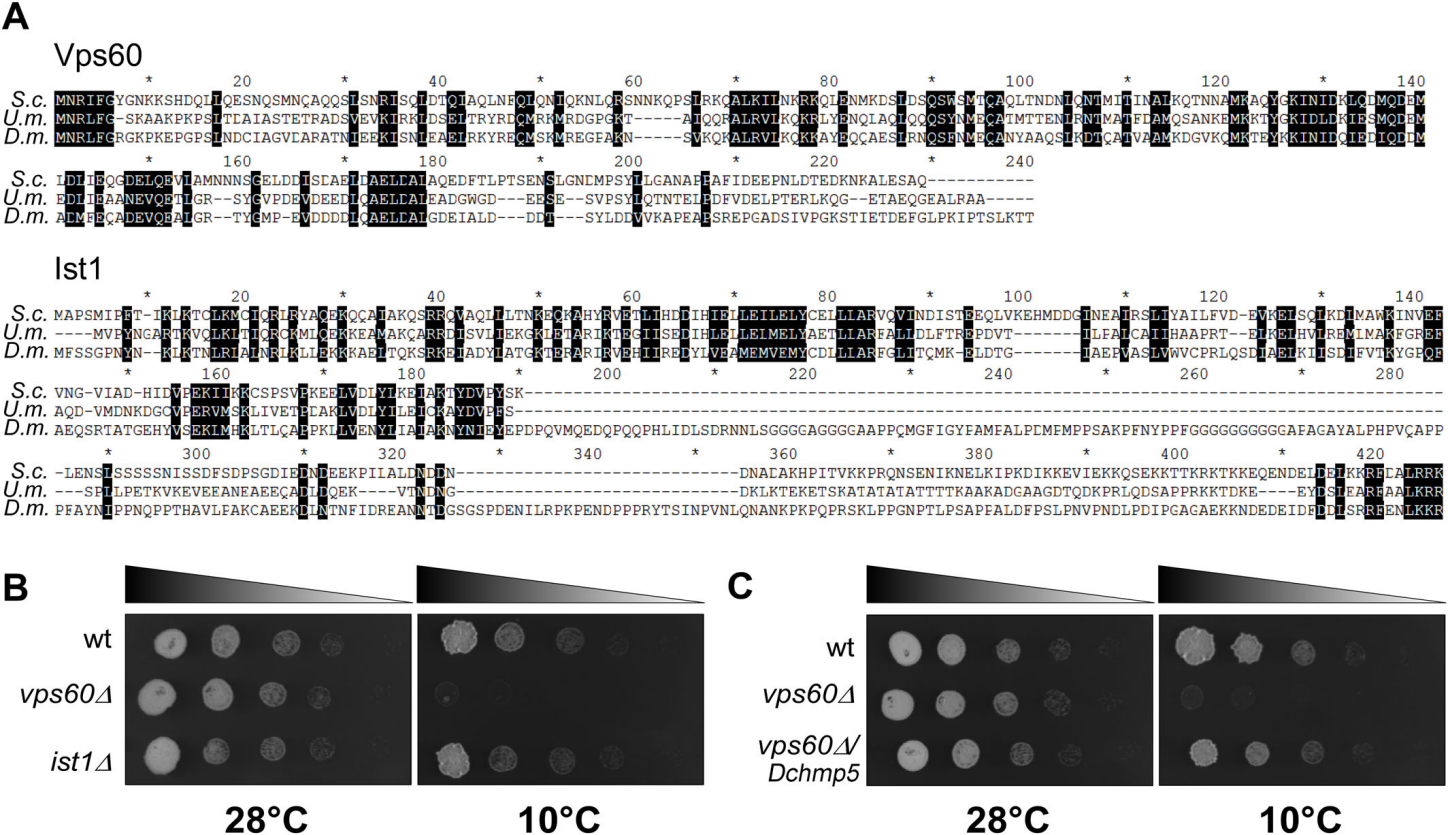
**Loss of *vps60/Dchmp5* causes a cold-sensitive growth defect in *U.*** ***maydis.*** (A) Sequence alignments of Vps60/DChmp5 and Ist1/DIst1 of *S.* *cerevisiae* (*S.c*), *U.* *maydis* (*U.m.*) and *D.* *melanogaster* (*D.m.*) (B) Growth of serial dilutions of the deletion mutants *vps60Δ* and *ist1Δ* at 28°C and 10°C. Growth of the *vps60Δ* mutant is almost completely abolished at 10°C. (C) Growth of serial dilutions of *vps60Δ* and the ectopic complementation *vps60Δ/DChmp5* at 28°C and 10°C. Ectopic expression of *DChmp5* rescued the cold sensitivity at 10°C. For B and C, wt corresponds to lab strain AB33.

One important question that arises from the analysis is whether the auxiliary ESCRTs confer a robustness to cold in other poikilothermic cells. A temperature-sensitive phenotype for Vps60 or Ist1 is not described for *Saccharomyces* *cerevisiae* (www.yeastgenome.org). We therefore generated *vps60* (*Dchmp5* ortholog) and *ist1* mutants in a related well-studied microorganism, the phytopathogen *U. maydis* (Fig. 8A). The ESCRT machinery was recently characterised in this model and shown to participate in long range transport of mRNAs besides its function in endosomal trafficking (Haag et al., 2017). We observed a similar cold-sensitive phenotype for *vps60* mutants in *U. maydis*; while colony growth was normal at 28°C, it was dramatically reduced at 10°C in comparison to wild type (Fig. 8B). This cold sensitivity was not observed in *ist1* mutants, which grew indistinguishable from wild type (Fig. 8B). Moreover, we found that *Dchmp5* can compensate for the function of *vps60* in *U. maydis* as expression of *Dchmp5* in the *vps60* mutants rescued the cold sensitivity to a large degree (Fig. 8C). This finding suggests that Vps60/Dchmp5 is required for cold tolerance of the ESCRT process in different poikilothermic cells.

## DISCUSSION

We here report the functional analysis of the auxiliary ESCRT-III proteins Chmp5/Vps60 and Ist1 in *Drosophila*. They represent the two sub-complexes that regulate the activity of the Vps4 complex. We found that the loss of *Dchmp5* function results in a cold-sensitive phenotype that includes the arrest of development in early third larval instar stage at 18°C and a slight over-proliferation of imaginal disc cells caused by weak ectopic activation of the Notch pathway. The activation of the Notch pathway in *Dchmp5* mutants was also observed during oogenesis (Berns et al., 2014) and therefore appears to be a general consequence of loss of *Dchmp5* function. The activation of the Notch pathway is a consequence of the endosomal defect, as it is suppressed by the removal of one functional copy of *shrub*. In addition, we found a defect in endosomal trafficking of Notch, Wg and Dl.

In contrast, loss of *Dist1* function causes no detectable phenotype despite the lethality at a low temperature. A similar difference in the severity of the phenotypes of *ist1* and *Dchmp5* was observed in yeast (Agromayor et al., 2009; Dimaano et al., 2008; Rue et al., 2008).

The phenotype of *Dist1* mutants differs from that recently reported for its binding partner in the auxiliary sub-complex CHMP1/Did2. It has been published that upon tissue specific depletion in the wing, margin or vein defect occurred, whereas general depletion resulted in lethality before the pupal stage (Crespo-Yàñez et al., 2018; Valentine et al., 2014). Thus, either the loss of function of Did2 is more deleterious for development than its partner Dist1, or the used RNAi constructs had off-target effects. In contrast to human Ist1 and similar to yeast, we did not find evidence for an involvement of Dist1 in cytokinesis. This additional function appears to have evolved in mammals. The observed phenotype of *Dist1* and *Dchmp5* is also not easily compatible with a role in Hh signalling, as it has been suggested recently for Did2 (Coulter et al., 2018; Matusek et al., 2014). The loss of Hh function results in severe defects in wing development (Zecca et al., 1995). Although we found that the development of *Dchmp5* mutants continuously expressed at 18°C arrest during the early phase of the third larval instar stage, the expression of Wg clearly indicated the formation of a wing pouch, as the single ring-like domain frames a considerable area. In addition, the *vg*QE, which is indirectly dependent on the activity of Hh and labels the wing pouch, is expressed (Kim et al., 1997). Moreover, the wing discs of *Dist1* mutant flies had a normal size and appearance, indicating that Hh signalling is not significantly affected. These facts support the notion that Did2 has an additional role that is independent of its role in the auxiliary ESCRT complex. Further support provides the finding that Did2 is involved in long-distance mRNA transport in *U. maydis* in addition to its established role in the endosomal pathway (Haag et al., 2017) and that it displays a stronger loss of function phenotype than Ist1 in yeast (Dimaano et al., 2008).

We found that *Dchmp5^9.2^ Dist1^51^* double mutants display a much stronger phenotype than the single mutants and resembles that of *vps4* mutants. This synergistic phenotype of the double mutant is not expected if the two genes act in the same pathway, if as in our case null mutants are used. Hence, the two genes and the two corresponding auxiliary sub-complexes act in different pathways required for the activity of Vps4.

At the first glimpse it is puzzling that we fail to observe activation of the Notch pathway in *Dchmp5^9.2^ Dist1^51^* double mutant cells. It might be due to the fact that we were able to analyse only mutant cells of flies shifted to 29°C where they display the weakest, but the only analysable phenotype. However, it has recently been reported that loss of *vps4* function does not cause uncontrolled activation of Notch signalling, but its inactivation (Legent et al., 2015). Our analysis suggests that the loss of function of both auxiliary ESCRT complexes phenocopies loss of *vps4* function. In the light of these results, the failure to detect Notch activation in the *Dchmp5^9.2^ Dist1^51^* double mutant cells is expected and suggests that strong accumulation of Shrub at the membrane of the ME suppresses Notch activation instead of inducing it, as observed in the absence of Shrub (Sweeney et al., 2006).

The cold sensitivity of the phenotypes of the two auxiliary ESCRT mutants was not described before. We observed a similar cold-sensitivity for *vps60* mutants in the phytopathogen *U. maydis*. The cold sensitivity could be partially rescued by introduction of *Dchmp5*, suggesting that the auxiliary ESCRTs confer robustness against cold for the ESCRT mediated process in very different poikilothermic organisms. In microorganisms, it has been found that modification of membrane composition is important to cope with cold stress (Guschina and Harwood, 2006). A major change is the increase in unsaturated fatty acids to maintain membrane fluidity at lower temperatures. Thus, one possible explanation might be that the auxiliary ESCRTs are required to adapt the ESCRT function to this change in membrane composition.

However, we observed that the reduction of the level of Shrub (*shrub* heterozygosity) rescues *Dchmp5* mutants completely and *Dchmp5 Dist1* double mutant to a considerable extent (pupal stage at 29°C, Fig. 5B). Moreover, *Dchmp5* mutant MEs contained more ILVs than its wild-type counterparts. These findings suggest that the activity of Shrub is elevated in the absence of functional auxiliary ESCRTs complexes and that this over-activation is deleterious at lower temperature. We found that also the reduction of the activity of *vps20*, resulted in a considerable rescue of *Dchmp5* mutants and that the overexpression of functional Myc-Shrub is deleterious to cells. In combination, these findings imply that it is probably the inappropriate accumulation of Shrub at the endosomal membrane, which causes its over-activation. Hence, a major task of the auxiliary ESCRTs is to prevent the deleterious accumulation of Shrub at the endosomal membrane at lower temperature in poikilothermic organisms, probably by enhancing the activity of Vps4.

## MATERIALS AND METHODS

### *Drosophila* genetics

The following fly stocks were used during this analysis. Mutants: *Dist1^51^ FRT2A* (this work), *Dchmp5^9.2^ FRT2A* (this work), *Dist^51^ Dchmp5^9.2^ FRT2A* (this work), *lgd^08^* (Gallagher and Knoblich, 2006), *shrub^4-1^ FRTG13* (Sweeney et al., 2006), *vps20^I3^* (Vaccari et al., 2009). UAS-constructs: *UAS Myc-shrub* (Troost et al., 2012), *UAS shrub-GFP* and *UAS shrub-*RNAi (Sweeney et al., 2006), *UAS vps4-*RNAi (VDRC #35126), *UAS Vps4-DN-Myc* (dominant negative variant, a gift from Robert Jaekel, Heinrich-Heine-University Düsseldorf), *UAS HA-Dchmp5* (this work). Others: *Gbe+Su(H)-lacZ* (Furriols and Bray, 2001), *NRE-pGR* (Housden et al., 2012), *wg-lacZ* (Kassis et al., 1992). *hedgehog*GAL4 (Tanimoto et al., 2000), *engrailed*GAL4 (BL), *tubulin*GAL80^ts^ (BL #7018), *vg*QE (a gift from Sean Carroll, R.M. Bock Laboratories). For the generation of mutant alleles, we used for *Dist1^51^*: y^1^ w*; P(EP)CG10103^G6550^/TM3, Sb^1^ Ser^1^ (BL #27211) and for *Dchmp5^9.2^*: y^1^ w*; P(EP)CG6259^G4547^/TM3, Sb^1^ Ser^1^ (BL #27160). Oregon R served as wild type. BL=Bloomington Stock Center; VDRC (Vienna Drosophila Resource Center).

### Generation of the alleles *Dchmp5^9.2^* and *Dist1^51^* and constructs

The alleles *Dchmp5^9.2^* and *Dist1^51^* were generated by imprecise P-element excision using the P-element insertion *P(EP)CG6259^G4547^* and *P(EP)CG10103^G6550^*, respectively. The created mutations were sequenced to define the breakpoints and deletion (see also Fig. 1A and Fig. S4A).

To generate *UAS HA-Dchmp5* we used the *Dchmp5* cDNA (DGRC, RE24819) as a template and fused a *SalI* restriction site, HA-tag and short linker encoding three glycines N-terminally and a *KpnI* restriction site C-terminally to the *Dchmp5* cDNA by PCR using following oligonucleotides: *GGAAGTCGAC ATGTACCCGTACGATGTTCCTGACTATGCGGGCGGAGGGATGAATCGCCTTTTTGGTCGTG* and *GTCCGGTACCCTATGTGGTCTTCAGCGATGT.* This DNA fragment was cloned into the multiple cloning site of *pUAST-attB* using the added *SalI* and *KpnI* restriction sites. *pUAST-attB-HA-Dchmp5* was inserted into the genomic landing site attP51C (Bischof et al., 2007).

### Clonal analysis, RNAi experiments and temporal expression of transgenes

The FLP/FRT clones (Xu and Rubin, 1993) were induced at first instar larval stage by applying a 90-min heat shock at 37°C.

The GAL4/UAS system (Brand and Perrimon, 1993) was used to overexpress transgene encoded proteins. GAL80ts was used to control the expression of *UAS vps4-*RNAi as well as *UAS Vps4-DN-Myc* for 24 h and UAS-Myc-Shrub for 48 h.

### Immunohistochemistry and microscopy

For the staining of wing imaginal discs standard protocols were used. The following antibodies were obtained from the Developmental Studies Hybridoma Bank: mouse anti-Wg 4D4 (1:50), mouse anti-Nextra (Notch extracellular domain) C458.2H (1:100), mouse anti-Delta C594.9B (1:100), mouse anti-Discs large 4F3 (1:500) and rat anti-DE-Cadherin DCAD2 (1:50). Rabbit anti-Rab7 (1:50) (Tanaka and Nakamura, 2008), rabbit anti-ßGal (1:1500) (Cappel), rat anti-HA 3F10 (1:500) (Roche) and mouse anti-Myc (1:125) (Santa Cruz Biotechnology). Rabbit anti-Shrub (1:125) (this work). Fluorochrome-conjugated antibodies were purchased from Invitrogen/Molecular Probes.

Images of wing imaginal discs were obtained at a Zeiss AxioImager Z1 microscope including a Zeiss Apotome and using Zeiss Axiovision. Larvae were imaged at a Zeiss Stemi 2000-C stereomicroscope.

### Generation of a polyclonal antibody against Shrub

GST-tagged Shrub was expressed in the *E. coli* strain C41 (Overexpress) and purified using glutathione Sepharose 4B medium (GE Healthcare). The verified Shrub was injected into rabbits (Cocalico Biologicals). The obtained Shrub antiserum was affinity purified and verified by western blotting (Fig. S1A).

### Transmission electron microscopy

Wing discs were fixed in 2.5% glutaraldehyde, washed in 100 mM phosphate buffer and postfixed in 2% osmium tetroxide in phosphate buffer for 1 h on ice. After contrasting en bloc in 2% uranyl acetate, the specimens were dehydrated in ethanol and embedded in araldite using acetone as an intermediate solvent. Thin sections were stained with 2% uranyl acetate and lead citrate. Sections were observed under an EM 902 (Zeiss) microscope at 80 KV.

For the statistical analysis of MVBs their perimeter was measured using ImageSP (TRS & SysProg). The obtained data were statistically analysed using Microsoft Excel and GraphPad Prism 7.0d.

### Quantitative analysis of MVBs

To quantify the ILV content, electron dense material within the lumen of photographed MVBs was measured by utilizing a self-written macro for the image processing software ‘Fiji’ (Schindelin et al., 2012). This analysis included the following steps. MVBs were manually outlined as regions of interest (ROIs) and stored for analysis. Each individual MVB was cropped first. To reduce noise and preserve boundaries as much as possible, a median filter of 2 pixel range was applied. MVBs were delimitated using the ‘Otsu’ algorithm (Otsu, 1979) and converted into a binary mask, highlighting areas of electron dense material of ILVs. The areas of all masked ILVs were summed up and divided by the area of the whole corresponding MVB, resulting in the quotient of ILV material per MVB in percent. Colorized overlays of estimated ILV areas with the original electron microscopy image were revisited for quality control after the analysis was finished. Area quotients of several MVBs are collected and plotted in a Box-Whisker diagram. The obtained data were statistically analysed using GraphPad Prism 7.0d.

### Standard molecular biology techniques and strain generation in *U. maydis*

For cloning, *E. coli* K-12 derivative Top10 (Life Technologies) was used. Transformation and cultivation of *E. coli* were performed according to standard protocols. Growth conditions and source of antibiotics for *U. maydis* are described elsewhere (Brachmann et al., 2004). For strain generation, protoplasts were transformed with linearised plasmid DNA. See the supplemental material for detailed information about strains, plasmids and oligonucleotides used in this study (Tables S1–S4). For ectopic expression of *Dchmp5*, the gene construct was inserted into the ip^S^-locus. All homologous recombination events were analysed via Southern blot (Brachmann et al., 2004). For all PCRs, genomic DNA from wild-type strain UM521 (*a1b1*) served as template.

### Test for cold sensitivity in *U. maydis*

Cultures were grown using conditions described elsewhere (Brachmann et al., 2004). Cultures were grown in CM medium supplemented with 1% glucose overnight to an OD_600_=0.5. For each strain, 50 µl of the overnight culture were serially diluted four times 1:5 with sterile H_2_O to a final OD_600_=0.0008. 2 µl of each dilution step were spotted onto culture medium plates supplemented with 1% glucose and incubated either over night at 28°C or for nine days at 10°C. Colony growth was documented with a LAS4000 imaging system (GE Healthcare).

## Supplementary Material

Supplementary information
