# Supplementary material for: The auxiliary ESCRT complexes provide robustness to cold in poikilothermic organisms
Source: Biol Open. 2019 Aug 14;8(9):bio043422. doi: 10.1242/bio.043422 (PMC6777356; doi:10.1242/bio.043422)
Supplement: Supplementary information [file biolopen-8-043422-s1.pdf]

Supplementary Figures

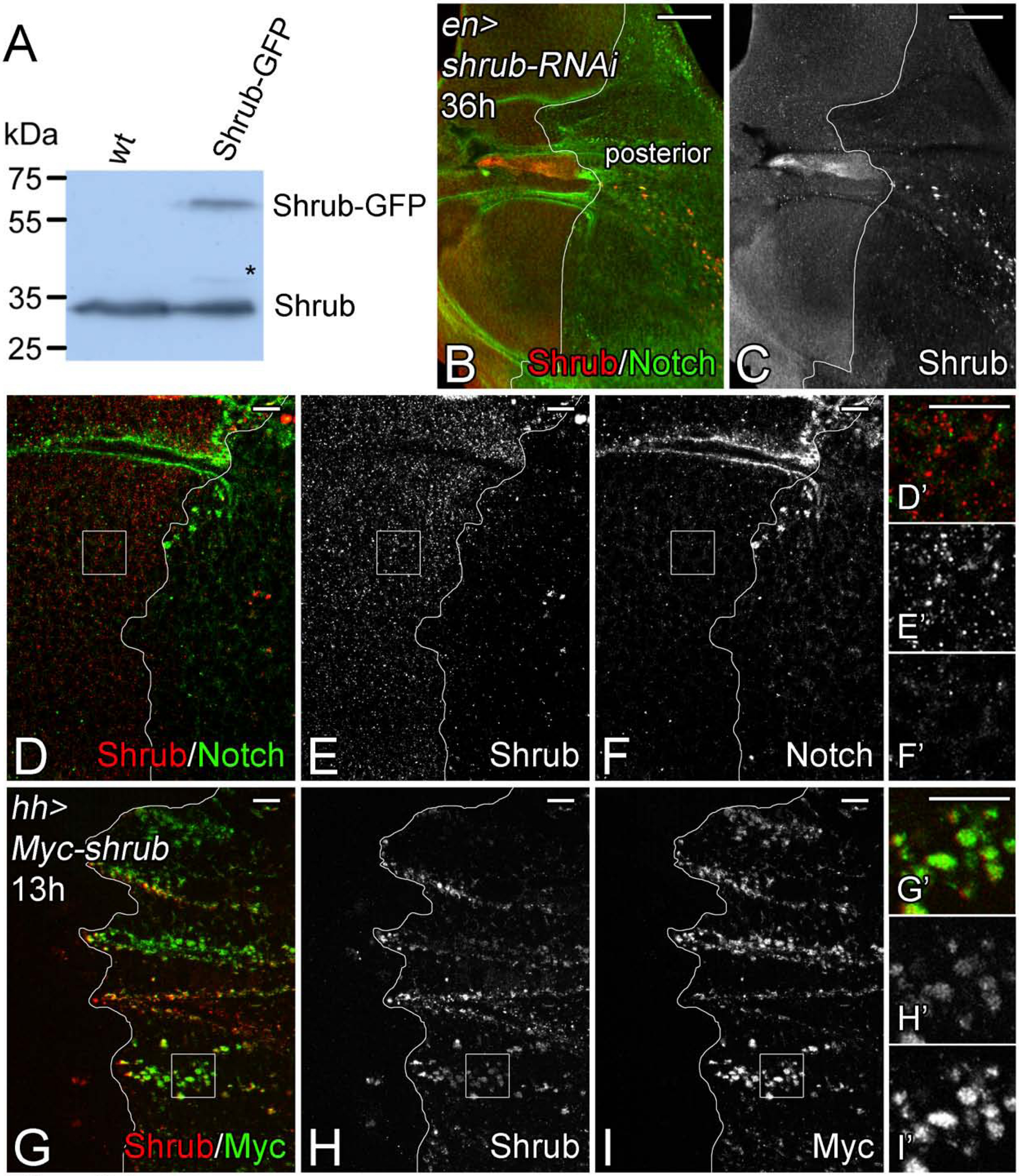

Fig. S1

Fig. S1. Characterisation of the Shrub antiserum.

(A) Western-blot of protein lysates of wild-type larvae and of larvae that expressed *UAS shrub-GFP* with *hs*GAL4. The anti-Shrub antibody specifically detects endogenous Shrub and the larger Shrub-GFP fusion protein. In addition, another weak band is detected upon over-expression of Shrub-GFP (asterisk), which probably constitutes a degradation product, since it is absent in the wild-type lane. (B-F) A wing imaginal disc where Shrub was depleted in the posterior compartment by expression of *UAS shrub*-RNAi for 36h using a combination of *en*GAL4 and *tubulin*GAL80^{ts}. The line labels the antero-posterior boundary. The antibody against Shrub detects a diffuse punctate pattern throughout the cytoplasm in the anterior control region. In the depleted posterior region, the Shrub signal is strongly reduced (C). High magnifications of the square region in (D-F) shown in (D'-F'). (G-I) Wing imaginal discs where Myc-Shrub was expressed for 13h using a combination of *hh*GAL4 and *tubulin*GAL80^{ts}. Anti-Shrub detects over-expressed Myc-Shrub at MEs. The A/P boundary is outlined in white. Magnified images of the square area in (G-I) shown in (G'-I'). Scale bars: (B-C) 50 µm; (D-I) 10 µm. At least 10 wing imaginal discs were analysed for each genotype.

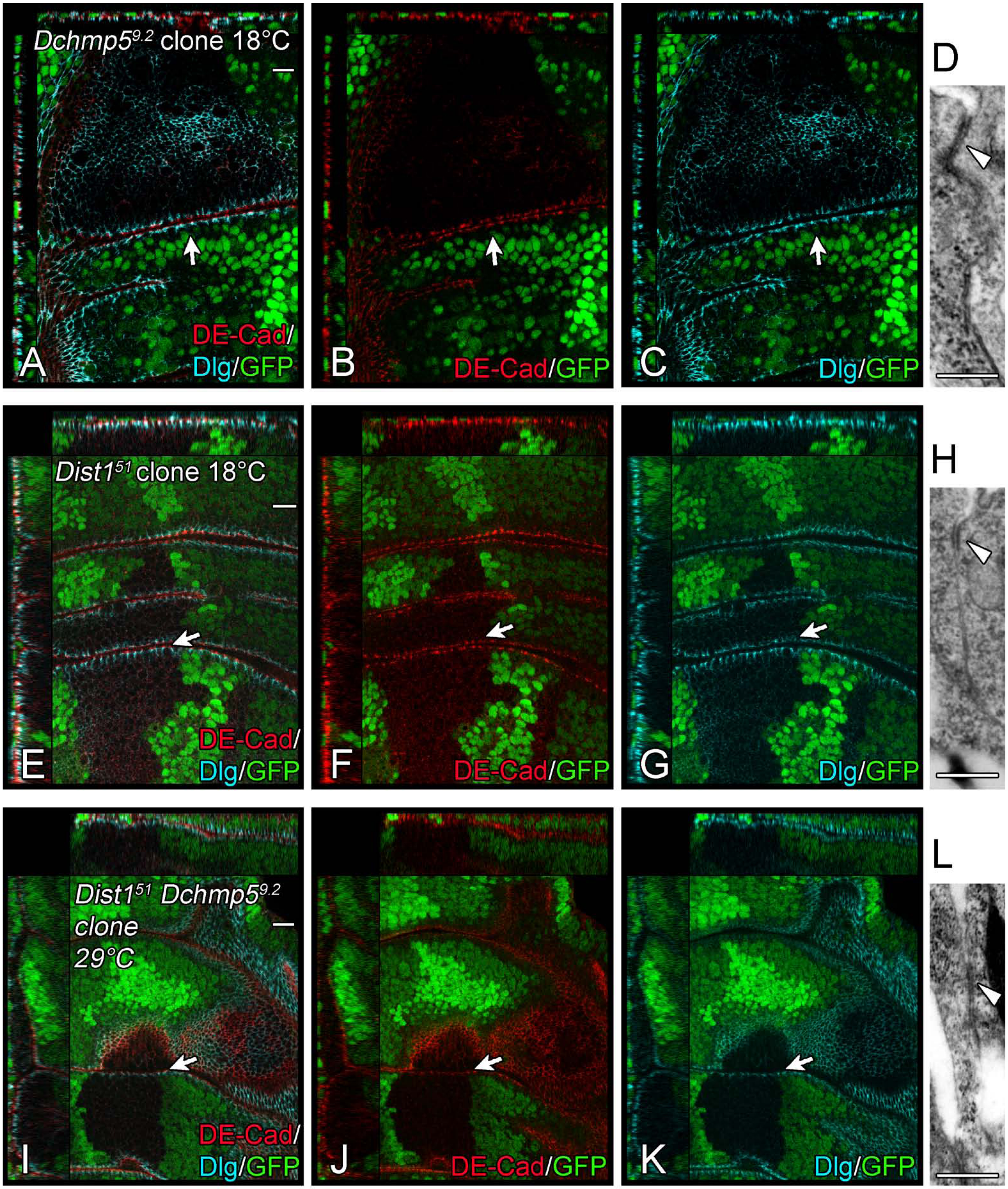
Dchmp5^9.2 clone 18°C
DE-Cad/
Dlg/GFP
A
B
DE-Cad/GFP
C
Dlg/GFP
D
Dist1^51 clone 18°C
E
DE-Cad/
Dlg/GFP
F
DE-Cad/GFP
G
Dlg/GFP
H
Dist1^51 Dchmp5^9.2
clone
29°C
I
DE-Cad/
Dlg/GFP
J
DE-Cad/GFP
K
Dlg/GFP
L

Fig. S2

Fig. S2. Apico-basal cell polarity is unaffected in *Dist1^{51}* and *Dchmp5$^{9.2}$* single and double mutants.

Clonal analysis of *Dchmp5$^{9.2}$* (A-C) and *Dist1^{51}* (E-G) single mutant (raised at 18°C) and *Dist1^{51} Dchmp5$^{9.2}$* double mutant (I-K) cells (raised at 29°C) in *Drosophila* wing imaginal discs. Clones are marked by the absence of GFP. The zonula adherens marker DE-Cadherin (DE-Cad) and the baso-lateral marker Discs large (Dlg) are normally localised in the single (A-C, E-G), as well as in *Dist1^{51} Dchmp5$^{9.2}$* double mutant cells (I-K). (D, H, L) TEM analysis of *Dchmp5$^{9.2}$* (D) and *Dist1^{51}* (H) single mutant and *Dist1^{51} Dchmp5$^{9.2}$* double mutant (L) cells. In all cases intact adherens junctions are observed (arrowhead), confirming that the apico-basal polarity is unaffected in these cells. Scale bars: (A-C, E-G and I-K) 10 µm; (D, H and L) 250 nm.

For clonal analysis at least 10 wing imaginal discs were analysed for each genotype. For ultra-structural analysis serval adherens junctions in at least three wing imaginal discs were analysed.

Biology Open • Supplementary information

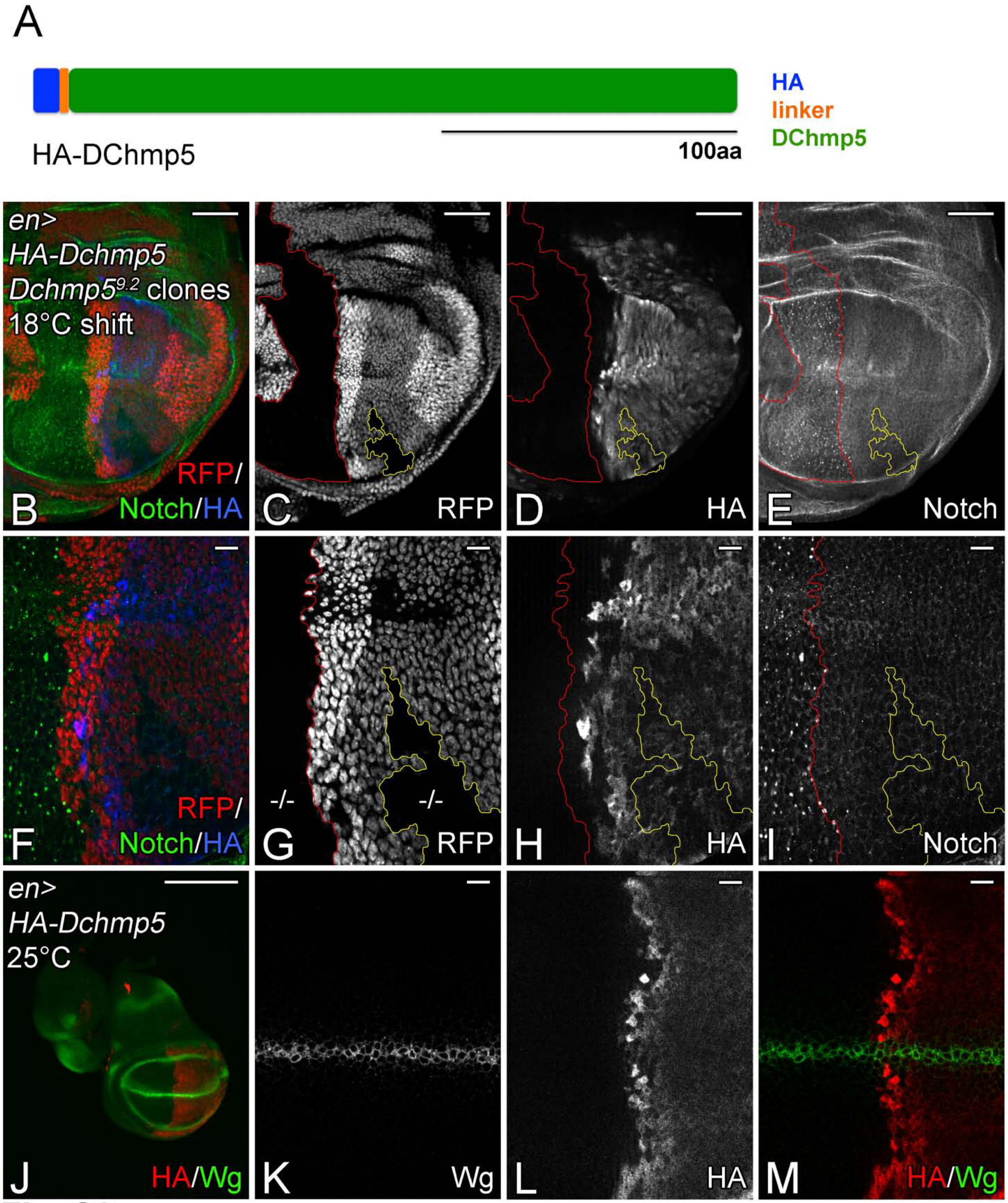
A
HA
linker
DChmp5
100aa
HA-DChmp5
en>
HA-Dchmp5
Dchmp5^9.2 clones
18°C shift
RFP/
Notch/HA
B
C
RFP
D
HA
E
Notch
-/-
-/-
F
G
H
I
en>
HA-Dchmp5
25°C
HA/Wg
J
K
Wg
L
M

Fig. S3

Fig S3. Overexpression of wild type DChmp5 in the mutant background reverses the Notch accumulation defects

Over-expression effects of HA-DChmp5 on the endosomal (B-I) and the activity of the Notch pathway (J-M). (A) Outline of the HA-DChmp5 construct. The N-terminal HA-tag (blue) is fused to the DChmp5 protein (green) by a short linker consisting of a triple glycine repeat (orange). (B-I) Imaginal disc bearing *Dchmp5*$^{9.2}$ mutant clones and expressing HA-DChmp5 in the posterior compartment with *en*Gal4. The flies were shifted to 18°C during the third instar larval stage. (C-I) A *Dchmp5*$^{9.2}$ mutant clone located in the anterior compartment is outlined in red, one in the posterior compartment in yellow. The enlargement of the MEs characteristic for *Dchmp5* mutant cells is observable in the anterior clone (outlined in red). This phenotype is suppressed in the posterior located clone (outlined in yellow) where the cells also express HA-DChmp5. (F-I) Magnifications of the disc shown in (B-E). (J-M) Continuous expression of HA-DChmp5 in a wild-type disc in the posterior compartment with *en*Gal4. No effect on the pattern of Wg expression can be observed. Scale bars: (J) 200 µm; (B-E) 50 µm; (F-I; K-L) 10 µm. At least 10 wing imaginal discs were analysed for each genotype.

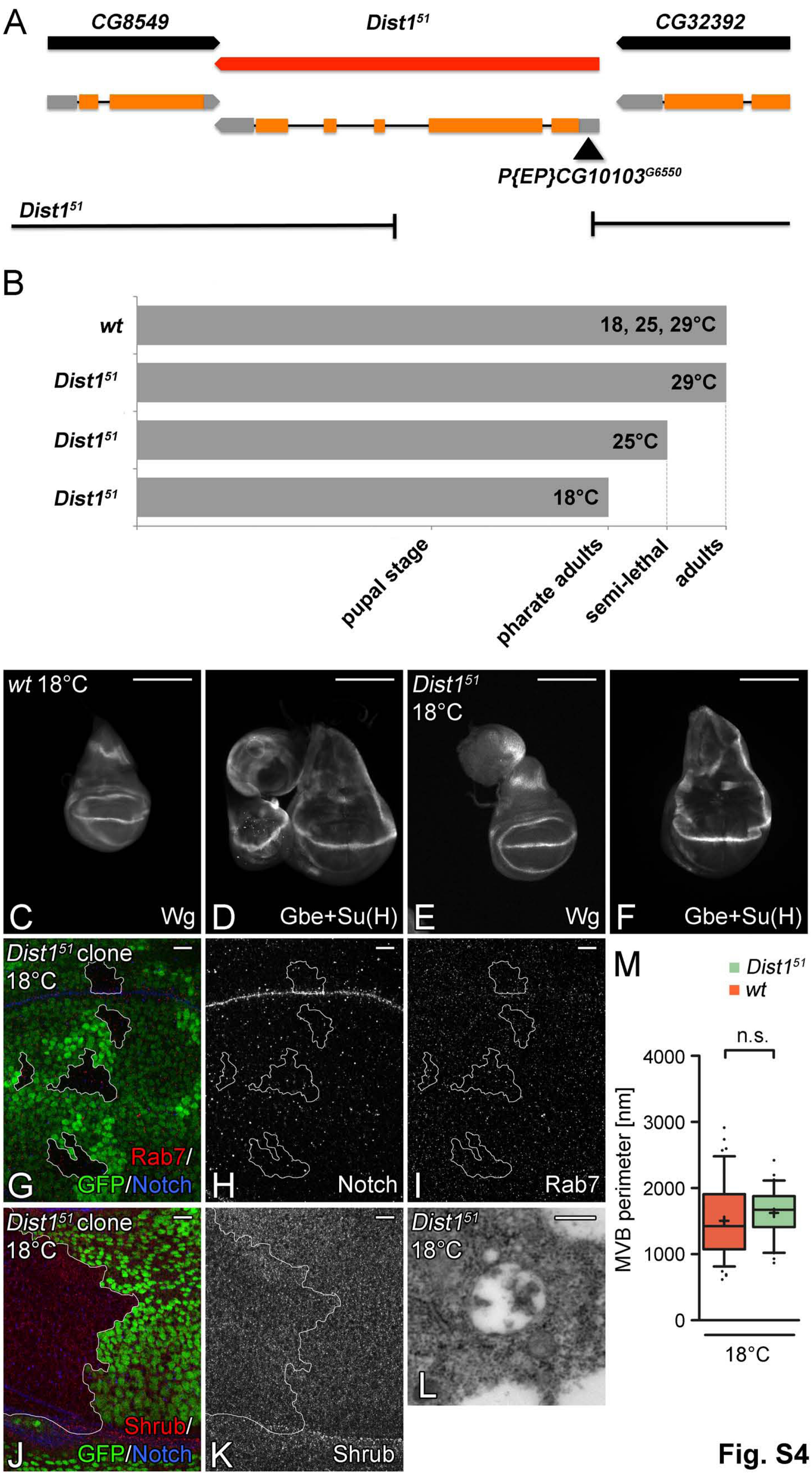

Fig. S4

Biology Open • Supplementary information

Fig. S4. Analysis of the function of *Dist1*.

(A) Representation of the genomic region of *Dist1* (red; adapted from FlyBase). The isoform A is shown below, exons in orange, UTRs in grey. The black triangle indicates the insertion site of the P-element *P(EP)CG10103*^{G6550}. The allele *Dist1*^{51} lacks 972 bp of the coding region including the transcriptional start ATG of all predicted isoforms.

(B) Summary of the time of death of *Dist1*^{51} null mutant flies at different temperatures.

(C-F) In comparison with wild-type wing imaginal discs (C, D) *Dist1*^{51} mutant discs (E, F) do not show any difference in the expression of Wg or Gbe+Su(H) even if raised at 18°C, indicating that the activity of the Notch pathway is normal in the *Dist1* mutants.

(G-K) Clonal analysis of *Dist1*^{51} mutant imaginal discs from flies constantly kept at 18°C. Clones are labelled through the absence of GFP and outlined in white. *Dist1*^{51} mutant cells contain normal MEs that are positive for Rab7 and/or Notch (G-I). No difference in the distribution of Shrub could be detected between mutant and wild-type cells (J, K). (L) The appearance of the MVBs of *Dist1* mutant cells is not different from that of wt cells. (M) Statistical analysis of the MVB perimeter in wild-type (red) and *Dist1*^{51} mutant (green) wing imaginal disc cells at 18°C showed no significant (n.s.) difference in size of MVBs (wt n=92; *Dist1*^{51} n=68; t-test: $p>0.1$) (Box-Plots: whiskers: 5-95 percentile; mean shown as "+",). Scale bars: (C-F) 200 µm; (G-K) 10 µm; (L) 250 nm. For analysis in fluorescence microscopy at least 10 wing imaginal discs were analysed for each genotype.

Biology Open • Supplementary information

Table S1: Description of strains used for analysis in *Ustilago maydis*

Strain	Relevant Locus	Progenitor strain	Short description
AB33	*B*	FB2	Inducible expression of b-heterodimer under control of *nar1* promotor (P_{nar1}: *bE2/bW1*). Filamentous growth inducible by switching nitrogen source (Brachmann et al., 2001)
AB33 vps60Δ	*vps60*	AB33	Deletion of *vps60*. ORF was replaced with a Hygromycin-resistance cassette.
AB33 ist1Δ	*ist1*	AB33	Deletion of *ist1*. ORF was replaced with a Hygromycin-resistance cassette
AB33 vps60Δ/Dchmp5	ip^S	AB33 vps60Δ	Ectopic complementation of *vps60Δ* in *U. maydis*. Codon-optimised *Dchmp5* from *Drosophila melanogaster* is ectopically expressed in the ip^S-locus

Table S2: Generation of strains used for analysis in *Ustilago maydis*

Strain	UMa*	Relevant genotype	Progenitor strain	Plasmid transformed	Reference
AB33	133	*a2* P_{nar}:*bW2 bE1*	FB2	pAB33	Brachmann et al. 2001
AB33 vps60	933	*vps60Δ*	AB33	pVps60Δ (pUMa1698)	This study
AB33 ist1Δ	1600	*ist1Δ*	AB33	pIst1Δ (pUMa2525)	This study
AB33 vps60Δ/Dchmp5	1802	*Vps60Δ/*ip^R*: Dchmp5*	AB33 vps60Δ	pP_{tef}-Dchmp5_cbxR (pUMa2754)	This study

Biology Open • Supplementary information

Table S3: Plasmids used for analysis in *Ustilago maydis*

Plasmid	pUMa*	Resistance cassette	Short description
pVps60Δ	1698	Hygromcin	Plasmid for deleting *vps60*. Cloned via Golden Gate cloning into pDest (Terfrüchte et al., 2013). The resistance cassette from pStorI_1hs is flanked by regions 0.8 kb upstream and 1 kb downstream of *vps60*. The regions were amplified by PCR with oligonucleotides oRL650/oRL651 and oRL652/oRL653 using genomic DNA from wild type strain UM521 as template.
pIst1Δ	2525	Hygromycin	Plasmid for deleting *ist1*. Cloned via Golden Gate cloning into pDest (Terfrüchte et al., 2013). The resistance cassette from pStorI_1hs is flanked by regions 1.2 kb upstream and 1.2 kb downstream of *ist1*. The regions were amplified by PCR with oligonucleotides oDD304/oDD305 and oDD306/oDD307 using genomic DNA from wild type strain UM521 as template.
pP_{tef}-DmVps60_cbxR	2754	Carboxin	Plasmid for targetet ectopic expression of *Dchmp5* in the ip^{S}-locus. *Dchmp5* was codon optimised for dicodon usage in *Ustilago maydis* and custom ordered from IDT (Leuven, Belgium). The gene is inserted between the constitutive promoter P_{tef} and the transcriptional terminator T_{nos}.

Biology Open • Supplementary information

Table S4: Oligonucleotides used for analysis in *Ustilago maydis*

Oligonucleotide	Sequence (5´-3´)	Comments
oRL650	GGTCTCGCCTGCAATATTGAAATCGAGCCGATTGGTC	Upstream flank of *vps60* (*u2*)
oRL651	GGTCTCCAGGCCGGTGAAAGAGTACGAGTAGATG	Upstream flank of *vps60* (*u3*)
oRL652	GGTCTCCGGCCGCGTCTTCTATTCAGATCCGC	Downstream flank of *vps60* (*d1*)
oRL653	GGTCTCGCTGCAATATTGCAGACGGTTCAACCGCG	Downstream flank of *vps60* (*d2*)
oDD304	GGTCTCGCCTGCAATATTGGCACCACCCTTGATCACC	Upstream flank of *ist1(u2)*
oDD305	GGTCTCCAGGCCCGCGTGGTAGAAGTGTTGAAGC	Upstream flank of *ist1* (*u3*)
oDD306	GGTCTCCGGCCTCCCGCACAGCAATTCCG	Downstream flank of *ist1* (*d1*)
oDD307	GGTCTCGCTGCAATATTACAGCGACCGAGGCCG	Downstream flank of *ist1* (*d2*)

Biology Open • Supplementary information
